# Review on Plant-Based Management in Combating Antimicrobial Resistance - Mechanistic Perspective

**DOI:** 10.3389/fphar.2022.879495

**Published:** 2022-09-29

**Authors:** Masita Arip, Malarvili Selvaraja, Mogana R, Lee Fang Tan, Mun Yee Leong, Puay Luan Tan, Vi Lien Yap, Sasikala Chinnapan, Ng Chin Tat, Maha Abdullah, Dharmendra K, Najwan Jubair

**Affiliations:** ^1^ Allergy and Immunology Research Centre, Institute for Medical Research, Ministry of Health Malaysia, Setia Alam, Malaysia; ^2^ Department of Pharmaceutical Biology, Faculty of Pharmaceutical Sciences, UCSI University, Cheras, Malaysia; ^3^ Immunology Unit, Department of Pathology, Faculty of Medicine and Health Sciences, Universiti Putra Malaysia, Serdang, Malaysia; ^4^ Narayan Institute of Pharmacy, Gopal Narayan Singh University, Jamuhar, India

**Keywords:** antimicrobial resistance, bacteria, virus, fungi, parasites, plants, secondary metabolites

## Abstract

Antimicrobial resistance (AMR) occurs when microbes no longer respond to any pharmacological agents, rendering the conventional antimicrobial agents ineffective. AMR has been classified as one of the top 10 life-threatening global health problems needed multilevel attention and global cooperation to attain the Sustainable Development Goals (SDGs) according to the World Health Organization (WHO), making the discovery of a new and effective antimicrobial agent a priority. The recommended treatments for drug-resistant microbes are available but limited. Furthermore, the transformation of microbes over time increases the risk of developing drug resistance. Hence, plant metabolites such as terpenes, phenolic compounds and alkaloids are widely studied due to their antibacterial, antiviral, antifungal and antiparasitic effects. Plant-derived antimicrobials are preferred due to their desirable efficacy and safety profile. Plant metabolites work by targeting microbial cell membranes, interfering with the synthesis of microbial DNA/RNA/enzymes and disrupting quorum sensing and efflux pump expression. They also work synergistically with conventional antibiotics to enhance antimicrobial effects. Accordingly, this review aims to identify currently available pharmacological therapies against microbes and AMR, as well as to discuss the importance of plant and secondary metabolites as a possible solution for AMR together with their mechanisms of action. All the information was obtained from government databases, WHO websites, PubMed, Springer, Google Scholar and Science Direct. Based on the information obtained, AMR is regarded as a significant warning to global healthcare. Plant derivatives such as secondary metabolites may be considered as potential therapeutic targets to mitigate the non-ending AMR.

## Introduction

Antimicrobial resistance (AMR) occurs when microbes such as bacteria, viruses, fungi and parasites transform over time and no longer respond to any pharmacological agents, making the infections resistant to treatments. This phenomenon increases the risk of disease spreading from one to another, leading to severe complications and increased deaths. Several infectious diseases, which include tuberculosis (TB), human immunodeficiency virus (HIV)/acquired immune deficiency syndrome (AIDS), diarrhoea, measles, pneumonia and malaria are known to be the major contributors to mortality in the global context (Cummins, 1999). Bacteria cause one proportion of these diseases while the larger proportions are actually by viruses and parasites which contribute to the relatively severe conditions. Diseases that have been associated with AMR include pneumonia, tuberculosis, diarrhoea, gonorrhoea, HIV/AIDS and malaria. Due to the insusceptibility of the drug-resistant pathogens against most of the conventional treatment options, it has imposed danger to the health of all the infected patients to a certain extent, especially to the patients who are vulnerable (e.g. elderly patients, patients with comorbidities, those who are immunocompromised or undergoing chemotherapy, *etc*.) (Dadgostar, 2019). Moreover, the emergence of AMR has led to consistently rising healthcare costs due to the increased morbidity, which requires prolonged hospital stays and drug usage in addition to increased mortality. According to the Centre for Disease Control and Prevention (CDC), it was proposed that approximately 50% of the antibiotics were prescribed unnecessarily in the US per se, which has been identified as one of the major causes of antibiotic resistance (Fair and Tor, 2014). Besides, some developing countries do not require a prescription for antibiotics due to the weak regulation enforcement, making the prescription-only medication available over the pharmacy counters. Thus, it may lead to inappropriate use of antibiotics for unindicated conditions, such as the common cold caused by viral infections, which will lead to the development of AMR in the long run (Fair and Tor, 2014). Apart from that, other factors such as environmental factors like contaminated water, poor sanitization, hygiene and awareness among the community are also considered drivers of AMR. Therefore, more control measures and enhanced enforcement legislation are required to create awareness and knowledge in the community (Ayukekbong et al., 2017).

On the other hand, the disability of antimicrobials in treating infections has led to the development of resistance among various strains of microorganisms. There are three types of AMR being described according to the number of antimicrobial drugs and the amount of the antimicrobial categories a microbe is resistant to, which are known as multidrug-resistant (MDR), extensively drug-resistant (XDR), and pandrug-resistant (PDR). According to the European Centre for Disease Prevention and Control (ECDC), MDR refers to the insusceptibility of the microbial isolate to at least one antimicrobial drug in three or more antimicrobial categories, while XDR refers to the insusceptibility of the microbial isolate to at least one drug in all but in two or less antimicrobial categories. PDR is defined as the insusceptibility of the microbial isolate to all drugs in all antimicrobial categories (Magiorakos et al., 2012). Genetic variation in the microorganisms is one of the leading causes of MDR occurrence over time (Cummins, 1999).

In clinical practice, as AMR persists as a disaster that causes elevated morbidity and mortality worldwide, it is being given attention to expedite the search for new and novel antimicrobials derived from plant derivatives. This approach is in line with one of the WHO action plan strategies Global Action Plan on Antimicrobial Resistance (GAP-AMR) in 2015, to escalate investments in diagnostic tools, new medicines, vaccines, and other interventions. There has been an increasing demand for novel antimicrobials to tackle the public health menace posed by AMR, but the discovery and development of new agents are now hindered due to the economic and regulatory obstructions (Ventola, 2015). Thus, this review aimed to provide comprehensive information on the list of resistant microbes, particularly bacteria and viruses and the potential of various plant derivatives as a treatment option to tackle AMR (Centers for disease control and prevention, 2017).

## Drug resistance in microbes

### Bacteria

Bacterial infections and high rates of resistance to antibiotics are common conditions that require attention globally. Antibiotic resistance is categorized according to its sources, respective modes of action, molecular structures and their spectrum of activity (broad-spectrum and narrow-spectrum) (Pancu et al., 2021). Various factors including inappropriate use and chronic over-prescription of antibiotics have caused the pathogens to acquire new resistance mechanisms toward the currently available antibiotics. This practice subsequently contributes to the inadequacy or failure of the standard treatments, leading to the extension of the disease period, higher cost of treatment and increased risk of death (Tanwar et al., 2014). The emerging infections frequently associated with this phenomenon include sepsis, diarrhoea, urinary tract infections and sexually transmitted infections. The list of WHO priority resistance pathogens with the current antibiotic treatment options are shown in Table 1. Due to the emergence of drug resistance, most of the currently available drugs are required to be taken in combination. Carbapenem-resistant *Acinetobacter* (CRA), carbapenem-resistant Enterobacteriaceae (CRE) and drug-resistant *Neisseria gonorrhoeae* are classified by the WHO as urgent threats globally. CRA causes pneumonia, bloodstream and urinary tract infections, whereas CRE frequently causes catheter-related infections. Both CRA and CRE synthesize carbapenemase enzyme that breaks down the carbapenem antibiotics and subsequently renders carbapenem ineffective. Moreover, they share the carbapenem resistance genes, which further limits the availability of effective antibiotics. The antibiotics indicated for carbapenem-resistant *Acinetobacter baumannii* infections are limited. The condition worsens as both CRA and CRE are commonly transmitted in the healthcare setting. Inappropriate cleaning and sanitization will cause an outbreak and increase the healthcare burden. WHO also reported that 50% of *N. gonorrhoeae* are resistant to at least one antibiotic, resulting in untreated gonorrhoea. As a result, the incidence of ectopic pregnancy, infertility, and cardiovascular and neurological problems increases (CDC, 2019).

**TABLE 1:** List of WHO priority drug-resistant bacteria and the currently available treatment options.

WHO priority drug-resistant bacteria	Antibiotic resistance	Priority category	Currently available treatment options	Reference
***Acinetobacter baumannii***	Carbapenem-resistant	Critical	Colistin (as colistimethate); 5 mg CBA/kg/day LD, followed by 5 mg CBA/kg/day divided into two to three doses	Viehman et al. (2014)
			Tigecycline; 100 mg LD followed by 50 mg q12h	
			Sulbactam (ampicillin/sulbactam); 3–9 g/day (9–27 g/day)	
			Rifampin^a^; 600 mg q12h or q24h	
			Vancomycin^a^; 10–15 mg/kg q12h, combination with colistin	
***Pseudomonas aeruginosa***	Carbapenem-resistant	Critical	IV Ceftolozane/tazobactam (ZERBAXA^®^); 1.5 g (1 g/0.5 g) q8h IV infusion over 1H	(Soliman et al., 2015; Doi, 2019)
**Enterobacteriaceae*,* ESBL-producing**	Carbapenem-resistant	Critical	Plazomicin; 15 mg/kg/day	Sheu et al. (2019)
			Eravacycline; 1 mg/kg q12h	
			Fosfomycin^a^; 4 g q6h or 8 g q8h	
***Enterococcus faecium***	Vancomycin-resistant	High	Quinupristin/Dalfopristin; 7.5 mg/kg q8h	Linden, (2002)
			Linezolid; 200 mg q12h	
***Staphylococcus aureus***	Methicillin-resistant, Vancomycin-intermediate and resistant	High	Vancomycin; 500 mg q6h	(Loomba et al., 2010; Rodvold and McConeghy, 2014)
			Linezolid; 600 mg q12h	
			Daptomycin; 4 mg/kg q24h	
			Tigecycline; 100 mg LD followed by 50 mg q12h	
			Telavancin; 10 mg/kg q24h	
			Ceftaroline; 600 mg q12h	
***Helicobacter pylori***	Clarithromycin-resistant	High	Combination	Mégraud, (2012)
			Bismuth subcitrate potassium; 40 mg	
			As one capsule q6h	
			Metronidazole; 125 mg	
			Tetracycline hydrochloride; 125 mg	
			Omeprazole; 20 mg q12h	
***Campylobacter *spp.**	Fluoroquinolone-resistant	High	Azithromycin; 500 mg q24h	Luangtongkum et al. (2009)
***Salmonellae***	Fluoroquinolone-resistant	High	Azithromycin; 500 mg q24h	Cuypers et al. (2018)
***Neisseria gonorrhoeae***	Cephalosporin-resistant, Fluoroquinolone-resistant	High	Combination	Kidd et al. (2014)
			PO Gemifloxacin; 320 mg + PO Azithromycin; 2 g	
			IM Gentamicin; 240 mg + PO Azithromycin; 2 g	
**Streptococcus pneumoniae**	Penicillin-non-susceptible	Medium	Levofloxacin, Gatifloxacin, Moxifloxacin	Ruhe et al. (2004)
**Haemophilus influenza**	Ampicillin-resistant	Medium	Cefepime, Chloramphenicol	Tristram et al. (2007)
***Shigella *spp.**	Fluoroquinolone-resistant	Medium	Ceftriaxone, Pivmecillinam, Azithromycin	Puzari et al. (2018)

a These agents are used in combination therapy and are not considered to be used alone.

Other than prophylaxis and treatment of infectious diseases, antibiotics have also been used in the veterinary field for growth promotion and feed efficiency improvement. Antibiotic residues in animal-derived food are detrimental to food safety and human health by causing issues such as antibiotic resistance, hypersensitivity reactions and aplastic anaemia in sensitive individuals (Ahmad et al., 2017; Ahmed et al., 2020a). Several approaches including chromatographic (e.g. high-performance liquid chromatography (HPLC), capillary electrophoresis (CE), mass spectrometry (MS)), microbiological, immunoassay (e.g. enzyme-linked immunosorbent assay (ELISA), chemiluminescence immunoassay (CLIA), radioimmunoassay (RIA), colloidal gold immunoassay (CGIA) and fluorescence immunoassay (FIA)) (Ahmad et al., 2017; Ahmed et al., 2020b), as well as biosensor methods have been employed to detect antibiotics residues for food safety and human health. A trend has been increasing for multi-residue detection methods incorporating miniaturization and portability (Lakew et al., 2022).

### Viruses

A never-ending recurrence of harmful viruses had significantly affected human health since the 18th century when the first tobacco mosaic virus was detected, leading to a 90% mortality rate worldwide. The continuous drug exposure and perpetual viral replication have led to the emergence of various resistant strains and endurance of infection despite treatment. Moreover, viral mutations that happen at high frequency in the viral genome during the replication process have also been the leading cause of the presentation of different variants among affected individuals (Zoulim, 2011). Antiviral resistance is also raising the burden in the immunosuppressed patient group. The resistance has developed to most antivirals including antiretroviral (ARV) drugs. The ARV drugs, including those from newer classes, are at risk of becoming partly or fully inactive due to the emergence of drug-resistant HIV. Since there are possibilities where the population receiving antiretroviral therapy (ART) may acquire resistance to HIV drug prescribed, appropriate and regular surveillance of the effectiveness of ART given would be benefial for the optimal selection and management of ART regimens (World Health Organization, 2020). The list of drug-resistant pathogens and the antiviral resistance drugs are included in Table 2.

**TABLE 2:** List of drug-resistant viruses and the currently available treatment options.

*Drug-resistant Viruses*	Antiviral resistance	Currently available treatment options	Reference
Influenza Virus	Amantadine-resistant, rimantadine-resistant	PO Oseltamivir; 75 mg BD for 5 days; or Inhaled Zanamivir; 10 mg (two 5-mg inhalations) BD for 5 days; or IV Peramivir: 600 mg IV infusion OD, given over 15–30 min	Uyeki et al. (2019)
	Oseltamivir-resistant	Inhaled Zanamivir: 10 mg (two 5-mg inhalations) BD for 5 days	Uyeki et al. (2019)
*Herpes Simplex Virus (HSV)*	Acyclovir-resistant	**Preferred therapy**	(Workowski and Bolan, 2015; Panel on Opportunistic Infections in Adults and Adolescents with HIV, 2019)
		IV Foscarnet; 40–80 mg/kg IV q8h until clinical resolution is attained; or **Alternative therapy (**≥ **21–28** **days):**	
		IV Cidofovir; 5 mg/kg once weekly; or Topical Trifluridine 1%; TDS; or Topical Cidofovir 1% gel; OD; or Topical Imiquimod 5% cream; three times a week; or Topical Foscarnet 1%; five times a day	
Human Immunodeficiency Virus *(HIV)*	**NNRTI + 2 NRTIs** - NNRTI ± 3TC or FTC-resistant - NRTI-resistant	DTG or BIC + 2 NRTIs (at least one fully active)^AI^; or Boosted PI + 2 NRTIs (at least one fully active)^AIII^ **;** or Boosted PI + INSTI^a^	Panel on Antiretroviral Guidelines for Adults and Adolescents., (2020)
	**Boosted PI + 2 NRTIs** - 3TC- or FTC-resistant	Continue same regimen^AII^ **;** or Another boosted PI + 2 NRTIs (at least one fully active)^AIII^ **;** or DTG or BIC + 2 NRTIs (at least one fully active; if only one of the NRTIs is fully active or if adherence is a concern, DTG is currently preferred over other INSTIs)^AIII^ **;** or Another boosted PI + INSTI^AIII^	Panel on Antiretroviral Guidelines for Adults and Adolescents., (2020)
	**INSTI + 2 NRTIs** - 3TC- or FTC-resistant	Boosted PI + 2 NRTIs (at least one fully active**)** ^AIII^ **;** or DTG or BIC + 2 NRTIs (at least one fully active**)** ^AIII^ **;** or Boosted PI + INSTI^AIII^	Panel on Antiretroviral Guidelines for Adults and Adolescents., (2020)
	**INSTI + 2 NRTIs** - EVG or RAL ± 3TC or FTC-resistant but susceptible to DTG	Boosted PI + 2 NRTIs (at least one fully active**)** ^AIII^ **;** or DTG BD or BIC (if HIV is sensitive) + 2 fully active NRTIs^BIII^ **;** or DTG^a^ BD or BIC (if HIV is sensitive) + a boosted PI^AIII^	Panel on Antiretroviral Guidelines for Adults and Adolescents., (2020)
Hepatitis B Virus *(HBV)*	Lamivudine-resistant	Switch to Tenofovir^b^; or Continue Lamivudine, add Tenofovir^b^; or Emtricitabine + Tenofovir	Terrault et al. (2018)
	Adefovir-resistant	Switch to Entecavir; 0.5 mg OD or Tenofovir^b^; or Continue Adefovir, add Entecavir	Terrault et al. (2018)
	Tenofovir-resistant	Switch to Entecavir; 0.5 mg OD; or Continue Tenofovir^b^, add Entecavir	Terrault et al. (2018)
	Entecavir-resistant	Switch to Tenofovir^b^; or Continue Entecavir; add Tenofovir^b^; or Emtricitabine + Tenofovir	Terrault et al. (2018)
	Telbivudine-resistant	Switch to Tenofovir^b^; or Continue Telbivudine, add Tenofovir^b^	Terrault et al. (2018)
	Multidrug-resistant (MDR)	Switch to Tenofovir; or Combine Tenofovir^b^ and Entecavir	Terrault et al. (2018)
*Hepatitis C Virus (HCV)*	Sofosbuvir-based and Elbasvir/Grazoprevir-resistant	Daily fixed-dose combination of 400 mg sofosbuvir/100 mg velpatasvir/100 mg voxilaprevir for 12 weeks^c^; or Daily fixed-dose combination of 300 mg glecaprevir/120 mg pibrentasvir for 16 weeks^d^ ^,^ ^e^.	American Association for the Study of Liver Diseases and the Infectious Diseases Society of America Glecaprevir/Pibrentasvir Treatment Failures, (2022)
	Glecaprevir/Pibrentasvir-resistant	Daily fixed-dose combination of 300 mg glecaprevir/120 mg pibrentasvir plus daily 400 mg sofosbuvir and weight-based ribavirin for 16 weeks; or Daily fixed-dose combination of 400 mg sofosbuvir/100 mg velpatasvir/100 mg voxilaprevir for 12 weeks; or For patients with compensated cirrhosis, addition of weight-based ribavirin is recommended for 12 weeks	American Association for the Study of Liver Diseases and the Infectious Diseases Society of America Sofosbuvir-Based and Elbasvir/Grazoprevir Treatment Failures, (2022)
	Sofosbuvir/Velpatasvir/Voxilaprevir-resistant	Daily fixed-dose combination of 300 mg glecaprevir/120 mg pibrentasvir plus daily 400 mg sofosbuvir and weight-based ribavirin for 16 weeks; or Daily fixed-dose combination of 400 mg sofosbuvir/100 mg velpatasvir/100 mg voxilaprevir plus weight-based ribavirin for 24 weeks	American Association for the Study of Liver Diseases and the Infectious Diseases Society of America Sofosbuvir-Based and Elbasvir/Grazoprevir Treatment Failures, (2022)

a LPV/r + RAL^AI^; other boosted PI (e.g., DRV) or INSTI (e.g., DTG)^AIII^

b Tenofovir Disoproxil Fumarate 300 mg daily or Tenofovir Alafenamide 25 mg daily.

c Genotype 3: Add weight-based ribavirin if cirrhosis is present.

d Not recommended for patients with prior exposure to an NS5A inhibitor plus NS3/4 PI regimens (eg. Elbasvir/grazoprevir).

e Not recommended for genotype 3 infection with sofosbuvir/NS5A inhibitor experience.

^AI^ Strongly recommended by the data obtained from randomized controlled trials = Strong; B = Moderate; C = Optional.

^AII^ Strongly recommended by the data obtained from well-designed non-randomized trials or observational cohort studies with long term clinical outcomes.

^AIII^ Strongly recommended by expert.

^BIII^ Moderately recommended by expert.

3TC, lamivudine; BIC, bictegravir; DRV, darunavir; DTG, dolutegravir; FTC, emtricitabine; EVG, elvitegravir; FTC, emtricitabine; INSTI, integrase strand transfer inhibitor; NNRTI, non-nucleoside reverse transcriptase inhibitor; NRTI, nucleoside reverse transcriptase inhibitor; PI, protease inhibitor; RAL, raltegravir; LPV/r, Lopinavir/ritonavir; DRV/r, Darunavir/ritonavir; Boosted PI, LPV/r + RAL or DRV/r.

HIV drug resistance (HIVDR) is a condition caused by a mutation in the genetic structure of HIV that influences the ability of a particular drug or combination of drugs to obstruct the replication of the virus. HIVDR is classified into three categories as acquired HIV drug resistance (ADR), transmitted HIV drug resistance (TDR) and pre-treatment HIV drug resistance (PDR). The development of ADR occurs when HIV mutations arise due to viral replications in individuals receiving ARV drugs (World Health Organization, 2020). TDR occurs in ARV drug-naïve people with no history of ARV drug exposure. It is usually detected when previously uninfected individuals are infected with virus with drug resistance mutations. PDR is either a TDR or ADR, or both that have been transmitted at the time of infection or may be acquired by prior ARV drug exposure (e.g. in women who received ARV drugs for the prevention of mother-to-child transmission of HIV, in people who have received pre-exposure prophylaxis, or in individuals reinitiating first-line ART after a period of treatment interruption without documented virological failures) (Koay et al., 2021). The administration of non-nucleoside reverse-transcriptase inhibitors (NNRTIs) from PDR among the adults initiating first-line therapy exceeded 10% in monitored countries in Africa, Asia and Latin America. Moreover, the increased prevalence of PDR among infants has led to increased resistance to NNRTI. In sub-Saharan Africa, more than 50% of newly diagnosed infants with HIV were found to inherent NNRTI-resistant virus. Due to the continuous rise in the level of HIV drug resistance and the emergence of more deadly viruses, WHO has taken the initiative to implement various drug-resistant programs (e.g. HIV drug resistance program) which aim to eradicate HIV infections and minimize the HIV-associated morbidity and mortality (World Health Organization, 2020).

Globally, the chronic Hepatitis B Virus (HBV) infection continues to be a great challenge to healthcare as more than 300 million people are affected by severe liver complications. Antiviral drug resistance causes an interruption in treating the condition and thereby leads to severe complications such as cirrhosis and hepatocellular carcinoma. Although numerous extensively studied antivirals with proven effectiveness are currently available, other factors such as genetic variation, the potency of a particular drug, viral fitness, cross-resistance and pharmacological barriers continue to influence the viral resistance to antiviral agents (Zoulim, 2011).

### Fungi

Emergence and a dramatic rise in fungal infections began to be noticed during the era of antibiotics development to treat bacterial infections. However, fungal infection has become a global burden mainly due to antifungal resistance. *Candida*, *Aspergillus*, *Pneumocystis* and *Cryptococcus* spp. are characterized as the most prevalent and invasive pathogenic fungi causing deaths worldwide (Arastehfar et al., 2020). The rise in fungal infections is also associated with the surge in other underlying conditions such as cancer, AIDS, diabetes mellitus and cystic fibrosis. *Candida auris* is one of the harmful fungi resistant to several antifungal agents, followed by *Aspergillus fumigatus*. If this situation persists, it may lead to treatment failures and prolonged hospital stays, which may opt for expensive treatment options. The treatment options for fungal infections are extremely limited. There are merely three classes of antifungal drugs available for the treatment of systemic fungal infections, including azoles that inhibit ergosterol biosynthesis by targeting lanosterol 14-α-demethylase, echinocandins that inhibit fungal cell wall biosynthesis by targeting β-1,3-D-glucan synthase, and polyenes that bind to ergosterol in the fungal cell membrane, forming large pores and leading to cell lysis (Revie et al., 2018). The limited arsenal of antifungal agents is further restricted by the emergence of acquired drug resistance among prevalent fungal pathogens, which is presumably due to the widespread use of the antifungals (Cowen et al., 2015). The list of drug-resistant pathogens and the antifungal resistance drugs are included (Table 3).

**TABLE 3:** List of CDC priority drug-resistant fungi and the currently available treatment options.

CDC priority drug-resistant fungi	Antifungal resistance	Priority category	Currently available treatment options	Reference
***Candida auris***	Azole-resistant	Urgent treats	**First line: Echinocandins**	Centers for Disease Control, (2020)
			IV Anidulafungin; LD 200 mg, MD 100 mg/day	
			IV Caspofungin; LD 70 mg/m^2^/day, MD 50 mg/m^2^/day	
			IV Micafungin; 100 mg/day	
			**Unresponsive to first line/persistent fungemia for >5** **days**	
			Switch/combination with IV Liposomal Amphotericin B; 5 mg/kg/day	
***Candida auris***	Multidrug-resistant, Pan-resistant	Urgent treats	Not well studied	(Zeidler et al., 2013; Fakhim et al., 2017; Eldesouky et al., 2018, 2020a, 2020b; Hager et al., 2018; Barat et al., 2019; De Oliveira et al., 2019; Wiederhold et al., 2019; Jaggavarapu et al., 2020; O’Brien et al., 2020)
			***In-vitro***	
			Flucytosine^a^ combinations with either Amphotericin B^a^, Azoles^a^ (Isavuconazole, Itraconazole, Posaconazole, Voriconazole), or Echinocandins^a^ (Anidulafungin, Caspofungin, Micafungin)	
			Colistin with Caspofungin	
			Micafungin and Voriconazole	
			Micafungin and Amphotericin B	
			Sulfamethoxazole and Voriconazole/Itraconazole	
			Lopinavir and Itraconazole	
			Aprepitant and Itraconazole	
			Suloctidil and Voriconazole	
			Ebselen and Anidulafungin	
			***In-vitro* and *in-vivo***	
			VT-1598	
			APX001A and APX001 (prodrug)	
			**Clinical trial (Phase III)**	
			PO Ibrexafungerp (formerly SCY-078) (CARES Study, NCT03363841)	
***Candida glabrata***	Fluconazole-resistant	Serious treats	**Symptomatic Candida Systitis/Symptomatic Ascending Candida Pyelonephritis**	(White, 2001; Pappas et al., 2016)
			Amphotericin B deoxycholate; 0.3–0.6 mg/kg/day for 1–7 days with or without PO Flucytosine; 25 mg/kg QID for 7–10 days	
			**Vulvovaginal Candidiasis**	
			Intravaginal Flucytosine (1 g) and Amphotericin B (100 mg) formulated in lubricating jelly base in a total 8 g delivered dose OD for 14 days	
***Candida glabrata***	Multidrug-resistant (MDR)	Serious treats	IV Liposomal Amphotericin B; 3–5 mg/kg/day	(Arendrup and Patterson, 2017; Denardi et al., 2017)
			***In-vitro***	
			Caspofungin and Voriconazole	
			Anidulafungin and Voriconazole/Posaconazole	
**Aspergillus fumigatus**	Azole-resistant	Watch list	**Invasive Pulmonary Aspergillosis (IPA)**	(Aigner and Lass-Flörl, 2015; Verweij et al., 2015; Novak et al., 2020)
			IV Liposomal Amphotericin B; 3–5 mg/kg/day	
			IV Voriconazole^a^; 6 mg/kg q12h for 1 day, followed by 4 mg/kg q12h	
			IV/PO Posaconazole; 300 mg BD on day 1, then 300 mg OD	
			IV Caspofungin^a^; 70 mg/day on day 1, then 50 mg/day thereafter	
			IV Micafungin^a^; 100–150 mg/day	
			Voriconazole/Posaconazole–Echinocandin combination	
			**CNS aspergillosis**	
			IV Liposomal amphotericin B; 5 mg/kg q24h	
			Addition of	
			High dose Posaconazole/Isavuconazole or Flucytosine	

a These agents are used in combination therapy and are not considered to be used alone.

Several mechanisms of antifungal resistance have been identified, such as alteration or overexpression of drug target, upregulation of multidrug transporters, activation of stress responses, and alterations in cellular pathways (Cowen et al., 2015; Revie et al., 2018). Erg11 in yeasts and Cyp51A/Cyp51B in moulds code for lanosterol 14-α-demethylase, an enzyme target of azole antifungals. Altered or overexpressed ERG11/cyp51A/cyp51B is one of the most common mechanisms of azole resistance and has been reported in azole-resistant isolates of *C. albicans* (Morais Vasconcelos Oliveira et al., 2021), *C. glabrata* (Redding et al., 2002), *C. tropicalis* (Vandeputte et al., 2005), and *A. fumigatus* (Handelman et al., 2021). Multidrug transporters such as ATP-binding cassette (ABC) transporters Cdr1 and Cdr2, as well as the major facilitator Mdr1, have been upregulated in azole-resistant isolates of many *Candida* species (De Micheli et al., 2002). Unlike azoles, echinocandins are not substrates for multidrug transporters. Thus, echinocandin resistance remains unaffected by these transporters and is primarily conferred by amino acid substitutions in Fks subunits of glucan synthase. Mutations and expression levels of FKS genes affect the levels of echinocandin resistance in *Candida* spp. and are associated with reduced clinical response (Shields et al., 2012). An engineered point mutation in the hot spot 1 region of the fks1 gene was also sufficient to confer resistance to echinocandins in *A. fumigatus* (Rocha et al., 2007).

### Parasites

Specifically in tropical countries, parasitic infections and resistance is regarded as one of the most severe and life-threatening condition. This is mainly due to the nature of the parasites and also the ineffectiveness of the vaccines. Most of the treatments available currently for parasitic infections are chemotherapeutic agents, in which their efficacies are often disrupted by the development of parasitic resistance. Therefore, it is vital to accurately identify the drug resistance mechanisms for appropriate selection of treatment options for parasitic infections, particularly in the case of malaria, leishmaniasis and toxoplasmosis (Ertabaklar et al., 2020). Malaria is a condition caused by five *Plasmodium* species (*P. falciparum*, *P. vivax*, *P. ovale*, *P. malariae* and *P. knowlesi*). Up to date, antimalarial drug resistance has been reported for *P. falciparum*, *P. vivax* and *P. malariae* with CQ resistance carrier (Pfcrt) gene mutation being identified as the cause of *P. falciparum* resistance. Chloroquine resistance is one of the severe forms which has caused an increase in malaria-related deaths. The treatment options currently used to treat resistant pathogens are summarized (Table 4).

**TABLE 4:** List of WHO priority drug-resistant parasites and the currently available treatment options.

WHO priority drug-resistant parasites	Antiparasitic resistance	Currently available treatment options	References
***Trichomonas vaginalis***	Metronidazole-resistant	PO Tinidazole	(Nyirjesy et al., 2011; Henien et al., 2019)
		Paromomycin Intravaginal cream	
***Plasmodium falciparum***	Multidrug-resistant (MDR)	Halofantrin; 8 mg/kg q6h for three doses	Bloland, (2001)
		Quinine; 650 mg TDS for 7 days	
		Artesunate; 4 mg/kg on day 1, followed by 2 mg/kg daily for 5–7 days	
		Artemisinin; 20 mg/kg on day 1, followed by 10 mg/kg daily for 5–7 days	
		Artemether; 4 mg/kg on day 1, followed by 2 mg/kg daily for 5–7 days	
		Atovaquone/proguanil; 1000 mg/400 mg daily for 3 days	
	Chloroquine-resistant	Mefloquine/Artesunate; 15 mg/kg (max. 1 g) followed by 10 mg/kg on day 3 for mefloquine/4 mg/kg artesunate daily for 3 days	Bloland, (2001)
		Sulfadoxine/Pyrimethamine+Artesunate; 25 mg/kg sulfadoxine/1.25 mg/kg pyrimethamine as single dose + 4 mg/kg artesunate daily for 3 days	
		Lumefantrine+Artemether; Semi-immune patients: 4 tablets/dose at 0, 8, 24 and 48 h (total: 16 tablets); Non-immune patients: 4 tablets/dose at 0 and 8 h, then BD for 2 more days (total: 24 tablets)	
		Amodiaquine; 25 mg/kg divided over 3 days	
		Sulfadoxine/pyrimethamine; 25 mg/kg sulfadoxine/1.25 mg/kg pyrimethamine as single dose	
		Mefloquine; 750 mg base to 1500 mg base depending on local resistance patterns	
***Plasmodium vivax***	Chloroquine-resistant	Halofantrine	Baird et al. (1995)

## Limitations or drawbacks of current therapeutic approaches in controlling AMR

It is crucial and demanding for the discovery of novel antimicrobials to overcome AMR. The use of old or new antibiotics and the development of resistance is unavoidable and is a never-ending phenomenon (Gould and Bal, 2013). Combination therapies confer advantages over the development of new antibiotics as the emergence of resistance often outpaces the development of new antimicrobial agents (Coates et al., 2011). A combination of two or more antimicrobial agents is one of the therapeutic approaches to combat AMR as combinations allow the inhibition of targets in the same or different pathways or inhibition of the same target in different ways, overcoming the inadequacies of monotherapy and decreasing the likelihood of resistance development (Worthington and Melander, 2013; Sheard et al., 2019). Despite this, the pharmacokinetic and pharmacodynamic interactions that result in adverse effects often limit the use of combination therapies in addition to the increased cost (Coates et al., 2020). Pharmacodynamic interactions occur when the pharmacological effect of one drug is affected by that of another drug in the combination. The interactions can be synergistic, additive, or antagonistic. Antagonistic drug interactions may occur such as that reported by the combined use of bacteriostatic and bactericidal drugs (Ocampo et al., 2014). For instance, among the 204 bacteriostatic/bactericidal antibiotic pairs, 61% showed antagonism in *E. coli*. Pharmacokinetic interactions affecting drug absorption, distribution, metabolism or elimination may alter the effective drug concentrations in blood and tissue, and accelerates resistance development due to exposure to sub-inhibitory antibiotic concentrations (Coates et al., 2020). Toxicity issues are another limitation of combination therapies. For instance, the combination of vancomycin and piperacillin-tazobactam has been significantly associated with acute kidney injury (AKI) than either vancomycin or piperacillin-tazobactam monotherapy (Al Yami, 2017; Scully and Hassoun, 2018).

## Potential of plants for AMR

Natural drug products and synthetic drugs differ significantly in terms of chemistry with natural products possessing a greater chemical diversity. The natural products have lesser nitrogen, phosphorus, sulphur and halogen, possess enhanced molecular complexity, scaffold variety, a greater stereochemistry, ring system diversity and carbohydrate content (Schmidt et al., 2008). Due to the presence of multiple and complex phytochemicals in natural plant extracts, the development of resistance is therefore reduced (Gupta and Birdi, 2017). The presence of multiple phytochemicals in a single plant allows the plant extracts to act through multiple pathways in combating infections. *Thymus vulgaris* L. [Lamiaceae] essential oil has been reported to act effectively against benzimidazoles-, macrocyclic lactones-, imidazothiazoles-resistant *Haemonchus contortus* and is able to target several stages of the parasite’s life cycle, which includes the ability to inhibit egg hatching, larvae motility as well as development (Ferreira et al., 2016). Apart from its ability to exert multiple mechanisms of action, natural plant extract and compounds can also be used together with conventional antibiotics to enhance the antimicrobial effect. This can be seen in the study conducted by Li *et al.* in which berberine, an alkaloid present in many plants, was observed to act synergistically with fluconazole against fluconazole-resistant *Candida albicans*. The authors proposed that fluconazole can increase the intracellular content of berberine probably by disrupting the fungal cell membrane, thus enhancing the action of berberine (Li et al., 2013).

## Plants secondary metabolites as potential therapies for AMR

Plants have existed in the universe for more than 400 million years, having a unique characteristic which allows them to protect themselves from infections. Every plant species are unique as they can produce structurally different intermediates and products of metabolism which are known as secondary metabolites. A wide variety of secondary metabolites such as tannins, terpenoids, alkaloids, and flavonoids found abundantly in plants have been reported to possess antimicrobial properties.

These active forms of secondary metabolites are biosynthesized through different steps during plant metabolism which are specific for each plant kingdom. In addition, they also diverge in quality and quantity for a given plant species as they grow at different locations (Jain et al., 2019). Plant secondary metabolites are small molecules with diverse structures having multiple functions including as a fuel for the plants, providing signalling and stimulatory or repressive action on enzymes in plant structure. The metabolites also function as a cofactor for the enzymes in catalytic activities and interact with other microorganisms for defence action (Jain et al., 2019). In contrast to primary metabolites, the absence of secondary metabolites does not affect the plant in the short term; however, it leads to a long-term impairment of the organism’s survival, fertility, and aesthetics. Secondary metabolites also showed a significant role in plant defence against herbivory and other interspecies defence. An extensive number of plant secondary metabolites are biologically active within the healthy plant, while some of them are inactive and present in certain precursors which activate and release these metabolites based on the infections acquired. Typically, the biological response of a plant is a combined effect of several secondary products that exist in the plant (Leicach and Chludil, 2014).

Secondary metabolites have been used for the treatment of various illnesses (Gupta and Birdi, 2017). They manifest various biological effects as antibiotic, antifungal, antiviral and antiparasitic. Studies have reported that the majority of the population from rural cities mostly with great diversity in flora continue to use and depend on traditional medicine or herbs to manage their health conditions. Thus, the search for medicinal plants with potential activity gains extra value as they are found abundantly. It was noticed that plants’ secondary metabolites represent the first-line treatment option desired by people, specifically traditional healers in treating infections and other ailments as they are effective and less expensive as compared to conventional medications (Maman Manzo et al., 2019).

A vast chemical diversity provided by plant-derived compounds may contribute to secondary metabolites’ antimicrobial mechanisms which may enable them to be considered as potential targets. Generally, the plants’ secondary products exhibit their beneficial effects in indirect ways by resembling endogenous metabolites, hormones, ligands, signalling molecules, or neurotransmitters (Bilal et al., 2017). Their mechanisms of action depend on their respective chemical structures and properties. They can act on the microbial cell in various ways, such as disruption of the efflux system of the cytoplasmic membrane, by interacting with membrane proteins (e.g. ATPases), affecting DNA/RNA synthesis and function, destabilization of the proton motive force with ion leaks, prohibition of enzyme synthesis, induction of coagulation of cytoplasmic constituents, and interference with the normal cell communication (quorum sensing) (Gorlenko et al., 2020).

The plants’ secondary metabolites are categorized according to several criteria, based on their chemical structure, composition by the presence or absence of nitrogen, solubility in organic solvents or water, and their biosynthetic origin. However, the most common classification is based on their biosynthetic pathway where the secondary metabolites are categorized into three large groups: terpenes (e.g. cardiac glycosides, carotenoids, sterols and plant volatiles), phenolic compounds (e.g. phenolic acid, lignans, coumarins, lignins, stilbenes, tannins, and flavonoids), and alkaloids (Mera et al., 2019). Around 33,000 terpenoids followed by 16,0000 alkaloids and 8182 flavonoids have been recognized in the dictionary of natural products. (Jain et al., 2019). Additionally, British Nutrition Foundation has added sulphur-containing compounds which include GSL, GSH, thionins, phytoalexins, lectins and defensins to the dictionary (Mazid et al., 2011).

### Terpenes

Terpenes or terpenoids are one of the largest groups of plant metabolites with huge structural diversity, consisting of 40,000 different compounds. The first member of this class was isolated from turpentine oil in the 1850s (Bohlmann and Keeling, 2008). Several plants and animals also generate terpenes for survival (Paduch et al., 2007) as their pharmacological and biological properties are required by all living organisms (Singh and Sharma, 2015). The terpenes are grouped based on the number of isoprene units molecules present in its structure, such as hemiterpenes (single isoprene unit), monoterpenes (two isoprene units), sesquiterpenes (three isoprene units), diterpenes (four isoprene units), sesterpenes (five isoprene units), triterpenes (six isoprene units), sesquarterpenes (seven isoprene units), tetraterpenes (eight isoprene units), as well as polyterpenes and norisoprenoids with long chains of isoprene units.

### Phenolics

Phenolics are the largest category of secondary metabolites found abundantly in plants. Phenols are categorized as flavonoids and non-flavonoids based on their chemical structures (Christensen and Brandt, 2007). Flavonoids are polyphenols containing 15-carbon atoms with two aromatic rings which are linked through a 3-carbon bridge (Abbas et al., 2017). Flavonoids are present on the skin layer of fruits and the epidermis layer of leaves and contribute to many essential functions like providing pigments to parts of plants, defence against radiation and protecting against diseases (Korać and Khambholja, 2011). The non-flavonoids consist of tannins, phenolic acids, hydroxycinammates, polyphenolics, stilbenes and their conjugated derivatives (Abbas et al., 2017).

### Alkaloids

Alkaloids are nitrogenous compounds originated from amino acids such as lysine, tyrosine, aspartic acid and tryptophan in which therapeutic activities were discovered 4000 years ago (Amirkia and Heinrich, 2014). They exhibit an enormous diversity in their origin, structure and pharmacological properties. Therefore, alkaloids are categorized based on their molecular precursors, structures, and botanical and biochemical origins. Alkaloids are classified as true alkaloids, protoalkaloids and pseudoalkaloids (Dey et al., 2020). True alkaloids originated from amino acids have nitrogen present within the heterocyclic ring. True alkaloids are very reactive in nature and have potent biological activity against cocaine, morphine and quinine (Roy, 2017). Protoalkaloids also contain a nitrogen atom in their structures, but not in the heterocyclic rings. L-tryptophan and L-tyrosine are the main precursors of this type of alkaloid. Pseudoalkaloids’ basic structure does not originate from amino acids but is linked with amino acid pathways by amination or transamination reaction. Caffeine, capsaicin and ephedrine are well-known examples of this group (Alves de Almeida et al., 2017).

## Plants with antimicrobial activity and their mechanisms of action

### Antibacterial activity

**FIGURE 1:**
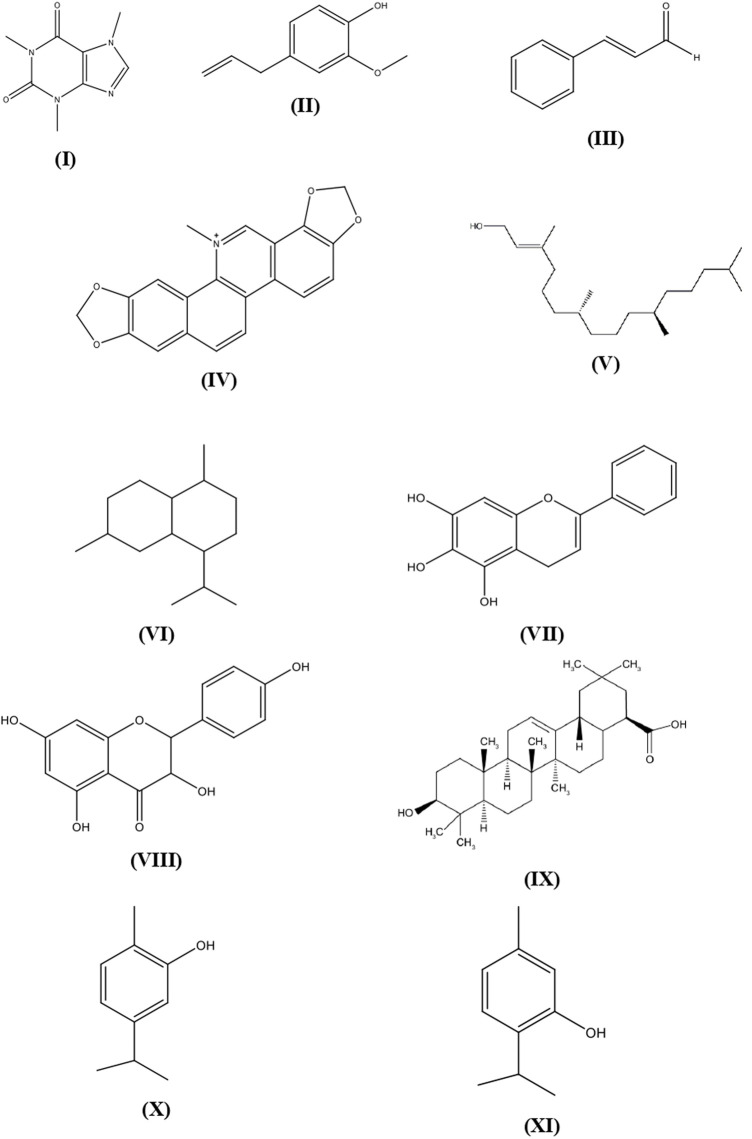
Plant secondary metabolites structures with antibacterial activity.

Various plants and their secondary metabolites have been reported for their antibacterial properties and the above are clearly summarized in Supplementary Table S1 with their mechanisms of action. The structures of several common metabolites are presented in Figure 1.

MDR pathogens share different mechanisms of action to escape from being eradicated by the currently available treatment options. Findings have reported that the plants’ secondary metabolites can effectively counteract the bacterial resistance through efflux pump inhibition (Kumar and Jandaik, 2016; Shriram et al., 2018). For instance, gallotannin (1,2,6- tri-O-galloyl-b-D-glucopyranose) obtained from *Terminalia chebula* Retz. [Combretaceae] fruits’ extract effectively inhibit MDR *Escherichia coli* with 2- to 4-fold decrease in minimal inhibitory concentration (MIC) of test antibiotics by inhibiting the ethidium bromide (EtBr) pump (Shriram et al., 2018). Similarly, methanolic extracts of *Acer saccharum* Marshall [Sapindaceae] showed inhibition of the efflux pump of *Pseudomonas aeruginosa* (ATCC 15692 and UCBPPPA14), *E. coli* (ATCC 700928) and *Proteus mirabilis* (HI4320) through EtBr efflux (Maisuria et al., 2015). In another study, tetrahydrosecamine, which is a piperidine alkaloid and strictanol, an indole alkaloid isolated from *Rhazya stricta* Decne. [Apocynaceae] displayed potent activity against MRSA, *E. coli* and *P. aeruginosa* by disrupting bacterial cell membrane (Khan et al., 2016). Although the exact mechanism remains unclear, there are several assumptions on how plant-derived efflux inhibitors work, such as canal occlusion, which is involved in evacuating the substrate by plant secondary metabolites. For instance, totarol, a diterpene from *Podocarpus totara* G.Benn. ex D.Don [Podocarpaceae], showed concurrent inhibition for NorA-pump in *Staphylococcus aureus* (Bhaskar et al., 2016). Furthermore, polyphenols may have direct attachment to the structural proteins of the efflux pump canal, leading to conformational alteration and preventing the elimination of the substance (Piddock et al., 2010). Secondary metabolites may have a synergistic effect on efflux pumps when combined with antibiotics. For example, combination of fluoroquinolones with reserpine from Rauvolfia vomitoria Wennberg [Apocynaceae] showed NorA efflux pump inhibition in S. aureus, while another combination consisting of norfloxacin with ferruginol from Sequoia sempervirens (D.Don) Endl. [Cupressaceae] causes blockage of ethidium bromide efflux (Stavri et al., 2007). It was suggested that terpenes enhance membrane disruption, coumarins reduce cell respiration and tannins work on microorganism membranes as well as bind to polysaccharides or enzymes, thus inducing inactivation (Anandhi et al., 2014). The degradation of genomic DNA due to endonuclease activation is considered an initial step in bacterial destruction. One study reported on the interference of glycosides and flavonoid compounds with bacterial DNA emphasized their ability to induce cleavage in the DNA fragments in bacteria like *E. coli*, *S. aureus* and *Klebsiella pneumoniae* (Anandhi et al., 2014). In another study, Palper and others reported that ATPase activity was markedly reduced after the binding of quercetin to the Gyr B subunit of *E. coli* DNA gyrase (Anandhi et al., 2014).

Bacterial biofilms are predominant features for bacteria to survive in an unfavourable environment. Biofilms are one of the causes of chronic, nosocomial, and medical device-related infections, which pose a great challenge to the healthcare system due to their high tolerance to antibiotics (Lou et al., 2019). Several plant-derived compounds showed anti-biofilm activity toward *P. aeruginosa*, *K. pneumonia* and staphylococcal biofilms, such as phenylpropanoids (e.g. eugenol and cinnamaldehyde), terpenoids (e.g. thymol and carvacrol), betulinic and ursolic acids, and alkaloids (e.g. berberine, indole, or chelerythrine) (Plyuta et al., 2013; Lou et al., 2019). The proposed mechanisms of plants’ secondary metabolites in inhibiting bacterial biofilm are through the disruption of intercellular communication, disturbance in cell-to-cell coaggregation, inhibition of cell mobility, inactivation of bacterial adhesins, or stimulation of bacteria dispersal (Silva et al., 2016).

In addition, quorum quenching is crucial to address AMR among pathogenic bacteria. It can be visualized in *P. aeruginosa* as it utilizes las and rhl quorum sensing (QS) systems for the synthesis of virulence factors, biofilm formation, gene expression and antimicrobial resistance (AMR) (Chan et al., 2015). An antimicrobial compound, eugenol, besides being able to inhibit the synthesis of procyanin and the swarming motility of P. aeruginosa, it was also found to reduce the expression of lasI and rhlI quorum sensing synthase genes that synthesize N-(3-oxododecanoyl)-L-homoserine lactone (3-oxo-C12-HSL) and N-butanoyl-L-homoserine lactone (C4-HSL) respectively. Both of these signal molecules regulate the transcription process of *P. aeruginosa* (Dekimpe and Déziel, 2009; Lou et al., 2019). On the other hand, methanolic extract of *Kalanchoe blossfeldiana* Poelln. [Crassulaceae] leaves inhibit the synthesis of virulence factors such as protease and pyoverdine through the interruption of N-acyl homoserine lactone (AHL)-mediated QS. Besides, the reduction of swarming motility, colony formation and biofilm formation were also observed (Sarkar et al., 2015). Urinary tract infections are commonly caused by *E. coli*, *P. mirabilis*, *P. aeruginosa* and *Serratia marcescens* (Hunjak et al., 2007). The pathogenesis of uropathogens is attributed to QS-mediated biofilm formation, making them relatively resistant to antibiotics (Jones et al., 2004). The production of biosurfactants, exopolysaccharides (EPS), swimming and swarming motility of bacteria are regulated by quorum sensing for the development of biofilm. Biosurfactant improves the swimming and swarming motility of pathogens, thereby accelerating the formation of biofilm (Caiazza et al., 2005). Furthermore, EPS maintains the rigidity of biofilm and protects the pathogens from exposure to antibiotics (Fux et al., 2005). Curcumin was found to inhibit the synthesis of EPS, swimming and swarming motility of *E. coli*, *P. mirabilis*, *P. aeruginosa* and *S. marcescens*. Confocal laser scanning microscopy (CLSM) demonstrated that curcumin disrupts biofilm’s structure and subsequently increases pathogens’ susceptibility to antibiotics. Curcumin also has the ability to inhibit alginate synthesis, the main constituent of EPS in *P. aeruginosa*. In addition, curcumin also interferes with the synthesis of biosurfactant (rhamnolipid) in *P. aeruginosa*, which reduces the occurrence of antimicrobial resistance. The anti-QS behaviour of curcumin towards *S. marcescens* is achieved through the decrease in virulence factor (prodigiosin) synthesis (Packiavathy et al., 2014).

As the available literature suggested, the primary target site for plant secondary metabolites action is the cytoplasmic membrane. Plant phytochemicals may affect cytoplasmic membrane structure and integrity, permeability or functionality in different ways (Radulovic et al., 2013). For instance, thymol, an aromatic monoterpene, interacts with the outer and inner cytoplasmic cell membranes by acting at the polar head group of the lipid bilayer, which will increase the membrane permeability for drugs (Walsh et al., 2003). Similarly, carvacrol, an isomer to thymol, interacts between acyl chains of the phospholipids, thereby increasing the membrane fluidity and higher membrane permeability (Ultee et al., 2000). Flavonoids also were found to affect membrane function, DNA synthesis, protein and RNA synthesis, but to a lesser extent. In a previous study, apigenin and quercetin together were found to inhibit DNA gyrase and β-hydroxyacyl-acyl carrier protein dehydratase activities (Cushnie and Lamb, 2005).

### Antiviral activity

**FIGURE 2:**
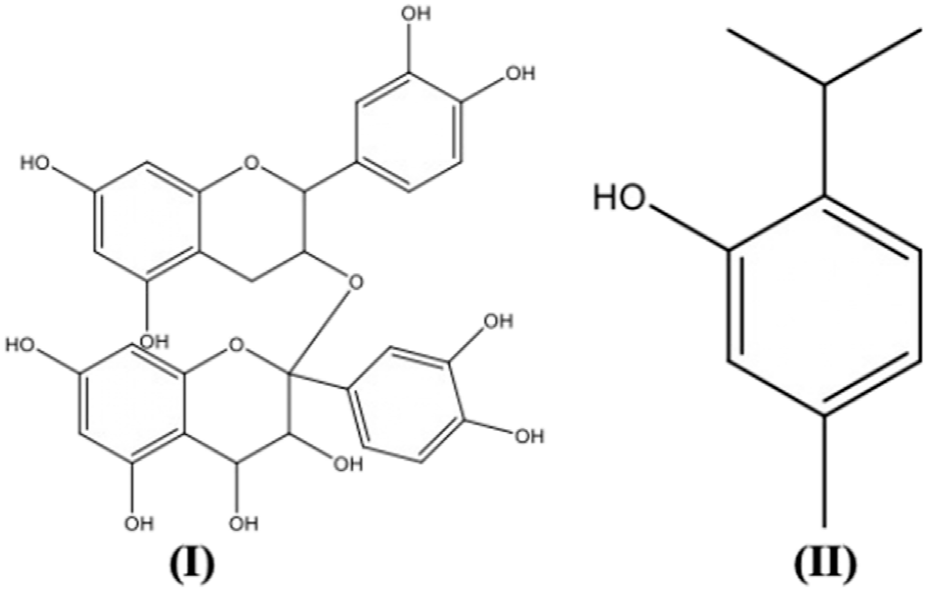
Plant secondary metabolites structures with antiviral activity.

Table 5 illustrates some selected phytochemicals and their antiviral mechanisms against drug-resistant viral infection. The structures of several common metabolites are presented in Figure 2. Hemagglutinin (HA) and neuraminidase (NA) are the major glycoproteins present in the influenza virus membrane. The viral HA plays an important role in the early stage of virus infection by mediating the binding of virions to sialic acid receptors on the plasma membrane and the release of viral genetic material into the cytoplasm *via* membrane fusion (Gamblin and Skehel, 2010). The polyphenolic compounds present in *Aronia melanocarpa* (Michx.) Elliott [Rosaceae], ellagic acid and myricetin have demonstrated good antiviral activity against various influenza virus subtypes, including an oseltamivir-resistant strain. The cross-reactivity of *A. melanocarpa* against oseltamivir-resistant H1N1 and rH5/IS06 viruses may be attributed to its anti-HA activity by non-specific masking of HA heads (Park et al., 2013). The essential oil vapours of *Citrus bergamia* (L.) [Rutaceae], *Eucalyptus globulus* Labill. [Myrtaceae] and the isolated compounds citronellol and eugenol, *Pelargonium graveolens* L'Hér. [Geraniaceae], *Cinnamomum verum* J.Presl [Lauraceae] and *Cymbopogon flexuosus* (Nees ex Steud.) W.Watson [Poaceae] were found to exhibit anti-influenza activity following short exposure times. A 100% activity was reported for the liquid phase of *C. verum*, *C. bergamia*, *C. flexuosus* and *Thymus vulgaris* L. [Lamiaceae] at the concentration of at 3.1 μl/ml, in which the antiviral activity may be attributed to the interaction with HA (Vimalanathan and Hudson, 2014). A study investigating the effect of anise oil, dwarf-pine oil and chamomile oil on acyclovir-sensitive and negative strains of HSV-1 reported high antiviral activity of these essential oils by interrupting viral adsorption. The ability of the essential oils to inhibit acyclovir-resistant HSV-1strains suggested the possible interaction of the essential oils with the virus before it enters the host cell as acyclovir acts *via* inhibition of DNA polymerase present in the cells (Koch et al., 2008).

**TABLE 5:** Plants and their secondary metabolites with reported antibacterial activity against vatious drug-resistant bacteria.

Plants	Reported secondary metabolites	Mechanism of action	Viruses	Assay method	Reference
*Aronia melanocarpa* (Michx.) Elliott [Rosaceae]	Ellagic acid (I)	Inhibit hemagglutinin protein	Oseltamivir-resistant influenza virus	Virucidal assay, MTT assay, hemagglutination inhibition assay, neuraminidase inhibition assay	Park et al. (2013)
	Myricetin (II)				
*Mentha pulegium* L. [Lamiaceae]	—	Prevent the synthesis of alpha or immediate early proteins or repressed the function of these proteins	Acyclovir-resistant HSV-1	MTT assay	Parsania et al. (2017)
*Zingiber officinale* Roscoe [Zingiberaceae] essential oil	—	Interfere virion envelope structures	Acyclovir-resistant HSV-1	Virucidal assay	Schnitzler et al. (2007)
*Thymus vulgaris* L. [Lamiaceae] essential oil	—	Interfere virion envelope structures	Acyclovir-resistant HSV-1	Virucidal assay	Schnitzler et al. (2007)
*Hyssopus officinalis* L. [Lamiaceae] essential oil	—	Interfere virion envelope structures	Acyclovir-resistant HSV-1	Virucidal assay	Schnitzler et al. (2007)
*Santalum album* L. [Santalaceae] essential oil	—	Interfere virion envelope structures	Acyclovir-resistant HSV-1	Virucidal assay	Schnitzler et al. (2007)
*Mentha × piperita* L. [Lamiaceae] essential oil	—	Block virus adsorption	Acyclovir-resistant HSV-1	Cytotoxicity assay, direct plaque assay	Schuhmacher et al. (2003)
*Carissa edulis* (Forssk.) Vahl	Lupeol (III)	—	Acyclovir-resistant HSV-1	Plaque inhibition assay, cell cytotoxicity assay, virus yield reduction assay	Tolo et al. (2010)
*Illicium verum* Hook.f. [Schisandraceae](Anise) oil	—	Interrupt herpes viruses adsorption	Acyclovir-resistant HSV-1	Plaque reduction assay	Koch et al. (2008)
*Pinus mugo* Turra [Pinaceae] (Dwarf-pine) oil	—	Interrupt herpes viruses adsorption	Acyclovir-resistant HSV-1	Plaque reduction assay	Koch et al. (2008)
*Matricaria chamomilla* L. [Asteraceae] (Chamomile) oil	—	Interrupt herpes viruses adsorption	Acyclovir-resistant HSV-1	Plaque reduction assay	Koch et al. (2008)

The antiviral activity of caffeine was associated with the inhibition of multiplication of both RNA and DNA viruses, such as HSV-1 and poliovirus. The inhibition of HSV-1 multiplication by caffeine occurs in the steps following the completion of viral DNA replication and nucleocapsid formation. Caffeine may also accelerate the degeneration of the infected cells (Koyama et al., 2008). Caffeic acid was reported to inhibit the replication of HSV-1 in a time-dependent manner in the early stage of the progeny infectious virus formation, probably by specific binding to the virus or molecules involved in replication before the viral DNA replication completes (Ikeda et al., 2011). A mechanism study revealed the inhibition of HCV replication by caffeic acid might be associated with the modulation of Keap1/Nrf2 interaction by upregulation of p62, causing the stabilization of Nrf2 and induction of HO-1, which would elicit the IFNα antiviral response (Shen et al., 2018). Besides, the inhibition of viral replication by epigallocatechin gallate (EGCG) involves the inactivation of important enzymes, such as RNA polymerase, protease, and reverse transcriptase that promote viral growth (Lipson et al., 2017). Viral proteases found in both enveloped and nonenveloped viruses have roles in catalysing peptide bond cleavage in viral polyprotein precursors or in cellular proteins, which are crucial for the completion of the viral infectious cycle (Steinkühler, 2008). Numerous plants have been reported to exhibit protease inhibitory activity against pathogenic viruses (Mandal et al., 2021).

Following virus replication, NA removes sialic acid from the surface of infected cells to release the newly synthesized virus for infection of more cells (Gamblin and Skehel, 2010). Punicalagin, an ellagitannin with strong anti-influenza activity, has demonstrated its primary mechanism of action by acting on the NA-mediated viral release, in addition to its inhibitory activity on the replication of influenza A and B viruses, including oseltamivir-resistant virus (NA/H274Y) (Li et al., 2021). A study on extracts of fifty medicinal plants from the tropical rainforests of Borneo against the H1N1 and H3N1 viruses revealed that all extracts acted through the inhibition of viral NA and only four extracts were found to inhibit hemagglutination. Furthermore, more than 90% reduction in viral adsorption and penetration were reported for samples which inhibited both hemagglutination and NA (Rajasekaran et al., 2013). The hemagglutination inhibition may be attributed to the presence of sialic acid-like component(s). The conventional drugs used for the management of influenza include NA inhibitors (NAI) such as oseltamivir and zanamivir as well as M2 blockers such as amantadine and rimantadine, and plant samples with anti-HA activity may be a potential solution for drug-resistant viruses (Rajasekaran et al., 2013).

To date, the emergence of a new coronavirus disease pandemic, namely COVID-19, has caused a serious threat to the public health worldwide, including those countries with good healthcare systems. COVID-19 is an infectious disease caused by a novel coronavirus (SARS-CoV-2 virus) with rapid transmission speed and different virulence levels among various newly developed coronavirus strains. Due to the rapidly increasing morbidity and mortality associated with COVID-19 infection, it has prompted the pharmaceutical industries all over the world to work on the development of vaccines and novel anti-COVID-19 drugs, such as Molnupiravir. RNA-dependent RNA polymerase (RDRP), 3CLPro and angiotensin-converting enzyme 2 (ACE2) have been identified as the major targets for anti-COVID-19 therapies. In general, RDRP regulates the transcription and replication of SARS-CoV-2, 3CLPro acts as the main viral proteinase that regulates the replication of SARS-CoV-2, while SARS-CoV-2 ACE2 plays a role in host cell attachment and membrane fusion. Based on the updated literature search, kaempferol derived from *Cassia senna* L. [Fabaceae], monoterpenes from *Zingiber officinale* Roscoe [Zingiberaceae] and S-allylcysteine from *Allium sativum* L. [Amaryllidaceae] demonstrated good affinity towards RDRP. Moreover, calcium elenolate derived from *Olea europaea* L. [Oleaceae], its monoterpenes and S-allylcysteine showed effective binding towards 3CLPro. In contrast, calcium elenolate, monoterpenes, dipropyl disulfide derived from *Allium cepa* L. [Amaryllidaceae], pelargonidin 3-galactoside from *C. senna* and thymoquinone from *Nigella sativa* L. [Ranunculaceae] demonstrated good affinity towards ACE2 (Mehmood et al., 2021). A peptide (GVITHDVSSAINRPQIGVVREFLTR) found in SARS-CoV-2 demonstrated greater affinity towards both kaempferol and anthraquinone derived from *Moringa oleifera* Lam. [Moringaceae] as compared to the standard drug, hydroxychloroquine (HCQ). Hence, these compounds with remarkable binding affinities towards RDRP, 3CLPro, ACE2 and the abovementioned peptide might be valuable leads for the development of novel SARS-CoV-2 antiviral drugs with substantial antiviral activity (Hamza et al., 2020).

### Antifungal activity

**FIGURE 3:**
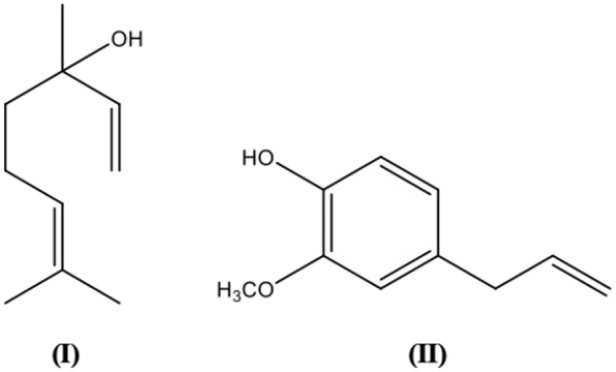
Plant secondary metabolites structures with antifungal activity.

Table 6 illustrates some phytochemicals and their antifungal mechanisms against drug-resistant fungal infection. The structures of several common metabolites are presented in Figure 3. Mechanisms involved in antifungal resistance include enzymatic drug inactivation, expression of efflux pumps and drug transporters, modifications in the drug target, alternative metabolic pathways and biofilm formation (Karpiński et al., 2021; Silva et al., 2021). As one of the most prevalent fungal pathogens causing fungal diseases in humans, the resistance of *Candida albicans* to antifungals is of concern. Concomitant use of berberine and fluconazole has been reported to demonstrate dose-dependent synergistic effect against drug-resistant *C. albicans in vitro*. The antifungal activity was attributed to the accumulation of berberine in *C. albicans*, particularly in the nucleus which caused cell cycle arrest and a remarkable change in the cell the cycle-related gene transcription (Li et al., 2013). Some studies have shown that *Candida tropicalis* may be resistance to fluconazole. The confirmed synergism of berberine and fluconazole suggested that berberine may be a potent agent to increase the efficacy of fluconazole in fluconazole-resistant *C. tropicalis*. The mechanisms involved include increased ROS generation, intracellular berberine accumulation, ergosterol reduction and the inhibition of efflux transporters (Shao et al., 2016).

**TABLE 6:** Plants and their secondary metabolites with reported antifungal activity against various drug-resistant fungi.

Plants	Reported secondary metabolites	Mechanism of action	Fungi	Assay method	Reference
*Berberis vulgaris* L. [Berberidaceae]	Berberine	(Work in synergism with fluconazole)	Fluconazole-resistant *C. albicans*	Cell cycle analysis by flow cytometry, intracellular BBR accumulation assay, gene expression analysis	Li et al. (2013)
		Disruptions of cell membrane function, structure and increase accumulation of berberine			
		Cell cycle arrest and DNA damage			
*Berberis vulgaris* L. [Berberidaceae]	Berberine	Increase ROS	Fluconazole-resistant *C. tropicalis*	Scanning electron microscope, FDA assay, Rh123 assay, HPLC	Shao et al. (2016)
		Inhibit efflux transporter			
		Reduce ergosterol synthesis			
*Aniba panurensis* (Meissn) Mez [Lauraceae]	Alkaloid	—	Drug-resistant strain of *C. albicans*	Broth microdilution assay	Klausmeyer et al. (2004)
-	Linalool (I)	Disruptions of cell membrane function and structure	Fluconazole-resistant *T. rubrum*	MIC, release of intracellular material using UV-VIS spectrophotometer, effect on morphogenesis *via* slide culture technique	De Oliveira Lima et al. (2017)
*Syzygium aromaticum* (L.) Merr. & L.M.Perry [Myrtaceae] (clove) essential oil	Eugenol	Disruptions of cell membrane function and structure	Fluconazole-resistant *A. fumigates*	MIC and MFC by broth macrodilution method, transmission electron microscopy	Khan and Ahmad, (2011)
			Fluconazole-resistant *T. rubrum*		
*Cinnamomum verum* J.Presl [Lauraceae] essential oil	Cinnamaledhyde	Disruptions of cell membrane function and structure	Fluconazole-resistant *A. fumigates*	MIC and MFC by broth macrodilution method, transmission electron microscopy	Khan and Ahmad, (2011)
			Fluconazole-resistant *T. rubrum*		
*Cymbopogon martini* (Roxb.) W.Watson [Poaceae]	Geraniol	Disruptions of cell membrane function and structure	Fluconazole-resistant *A. fumigates*	MIC and MFC by broth macrodilution method, Transmission electron microscopy	Khan and Ahmad, (2011)
			Fluconazole-resistant *T. rubrum*		
*Origanum vulgare* L. [Lamiaceae] essential oil	Carvacrol	Disruptions of cell membrane function and structure	Azole non-susceptible *C. neoformans*	Broth microdilution method	Scalas et al. (2018)
-	Teasaponin	Inhibit hyphae and biofilm formation by reducing cAMP level	Azoles multi-resistance *C. albicans*	MIC with broth microdilution method, inhibition of filamentation with modified broth microdilution method, XTT reduction assay, microscopic observation	Li et al. (2020)
*Tribulus terrestris* L. [Zygophyllaceae]	Saponin	—	Fluconazole-resistant *C. albicans*	MIC with microbroth dilution method	Zhang et al. (2005)
	TTS-12				
	TTS-15		Inherently-resistant *C. neoformans*		

Disruptions of cell membrane function and structures are among the other mechanisms by which some plants’ bioactive compounds exert their antifungal activity, as shown by monoterpene linalool, eugenol, cinnamaldehyde, geraniol and carvacrol (Khan and Ahmad, 2011; De Oliveira Lima et al., 2017; Scalas et al., 2018). A study on the clinical isolates of 5-fluorocytosine and fluconazole-resistant *Trichophyton rubrum* revealed that both linalool and ketoconazole significantly inhibited mycelial growth, conidial germination and conidiogenesis, induction of micro-morphological changes, and the induction of intracellular material leakage (De Oliveira Lima et al., 2017). The high fungistatic activity demonstrated by the sterol alkaloid tomatidine may be due to its involvement in the ergosterol pathway as shown by the upregulation of ergosterol genes in tomatidine-treated *C. albicans* (Dorsaz et al., 2017). Numerous studies have reported anti-cryptococcal activities of plant extracts and essential oils which may be attributed to the induction of fungal morphological changes, ergosterol synthesis inhibition, cell division interruption, reduced activity of enzymes such as laccase and urease as well as inhibition of biofilm formation (Silva et al., 2021). Besides, biofilm production may inhibit the activity of antifungals and contribute to drug tolerance or resistance. A recent review reported that 69 compounds derived from plants had exhibited activity against biofilms produced by *Candida* spp., either by acting on the formation and/or mature biofilms, suggesting the potential of plant preparations and bioactives as an alternative to antifungals (Silva et al., 2021).

### Antiparasitic activity

**FIGURE 4:**
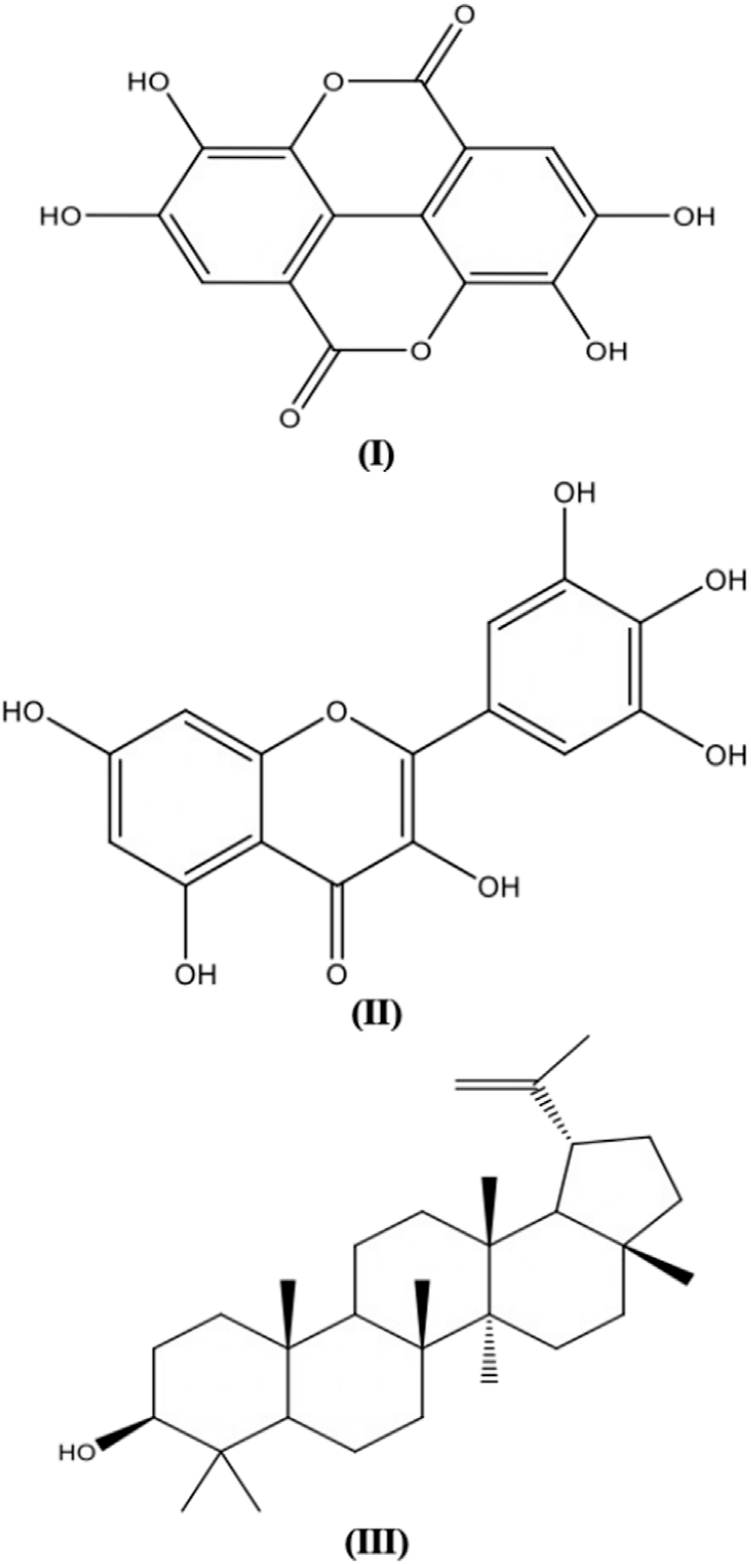
Plant secondary metabolites structures with antiparasitic activity.

Table 7 illustrates some phytochemicals and their antifungal mechanisms against drug-resistant fungal infection. The structures of several common metabolites are presented in Figure 4. The general antiparasitic-resistance mechanisms include hiding in sanctuary sites, modification of drug uptake systems, membrane composition alteration, drug inactivation, drug excretion, suppression or loss of drug activation and decreased drug-target interaction (Ouellette and Ward, 2003). Irreversible DNA damage due to the formation of covalent bonds between DNA alkylating compounds such as aristolochic acid, cycasin, furanoquinoline alkaloids, furanocoumarins, pyrrolizidine alkaloids and ptaquiloside, with DNA bases have cytotoxic and antiparasitic activities. DNA intercalation by berberine, sanguinarine, furanoquinoline alkaloids, emetine, beta-carboline alkaloids, anthraquinones and furanocoumarins may also contribute to DNA damage in the parasites (Wink, 2012).

**TABLE 7:** Plants and their secondary metabolites with reported antiparasitic activity against various drug-resistant parasites.

Plants	Reported secondary metabolites	Mechanism of action	Parasites	Assay method	Reference
*Warburgia ugandensis* subsp. Ugandensis [Canellaceae]	Polygodial	Inhibits mitochondrial ATP synthesis	Ivermectin-, Benomyl-, Aldicarb- and Levamisole- resistant *C. elegans*	Antihelmintic assay, MTT assay	Liu et al. (2018)
*Psidium guajava* L. [Myrtaceae]	Hydrosoluble compound	Reduce locomotion	Levamisole-resistant *C. elegans*	Antihelmintic assay	Piña-Vázquez et al. (2017)
		Induce paralysis			
		Inhibit egg laying			
*Tagetes erecta* L. [Asteraceae]	Hydrosoluble compound	Reduce locomotion	Levamisole-resistant *C. elegans*	Antihelmintic assay	Piña-Vázquez et al. (2017)
		Induce paralysis			
		Inhibit egg laying			
*Terminalia leiocarpa* (DC.) Baill. [Combretaceae]	Ellagic acid	Act on nervous system and pharyngeal muscle cells	Albendazole-, Levamisole-, Ivermectin-resistant *C. elegans*	Worm viability with binocular microscope, *in-vitro* screening assay	Ndjonka et al. (2014)
*Acacia nilotica* (L.) Willd. ex Delile [Fabaceae]	Proanthocyanidins (I)	Induce paralysis in worms	Albendazole-, Levamisole-, Ivermectin-resistant *C. elegans*	*In-vitro* assay	Vildina et al. (2017)
		Induce nematotoxicity			
*Zingiber officinale* Roscoe [Zingiberaceae] with apple cider vinegar	—	—	Spectrazole-, Fenbendazole-, Ivermectin-, Albendazole-resistant *Trichostrongylous* spp*.*, *Ostesteragia* spp., *Haemonchous* spp. and *Trichostrongylous* spp.	Fecal egg count reduction test (FECRT)	Hayajneh et al. (2019)
*Thymus vulgaris* L. [Lamiaceae] essential oil	Thymol (II)	Inhibit egg hatching	Benzimidazoles-, Macrocyclic lactones-, Imidazothiazoles-resistant *H. contortus*	Egg hatch test, Larvae motility assay, Larvae development test	Ferreira et al. (2016)
		Inhibit larvae motility and development			

Other than its implementation in anticancer therapy, topoisomerase inhibitors which interfere with the replication process leading to parasite death may also be employed as antiparasitic. For instance, a reduction in the viable *Trypanosoma cruzi* was found after treatment with baicalein and evodiamine (Lacombe et al., 2014). Synergistic effect of polygodial and warburganal with alpha-linolenic acid against *Caenorhabditis elegans* nematodes was reported, and *C. elegans* which demonstrated resistance against major anthelmintics did not show significant resistance to polygonial. The underlying mechanism of polygonial against *C. elegans* may be attributed to its dose-dependent inhibition of mitochondrial ATP synthesis (Liu et al., 2018). The water-soluble compounds present in the leaves of *Psidium guajava* L. [Myrtaceae] and flower-heads of *Tagetes erecta* L. [Asteraceae] were reported to significantly paralyze *C. elegans* (wild-type and lemivasole-resistant) with a mechanism different to that of lemivasole. These extracts also inhibited egg-laying behaviour of the parasites and the oviposition inhibition may be resulting from the blocking of the Gαq and/or stimulation of the Gα0 pathways (Piña-Vázquez et al., 2017). The high potency of ellagic acid with LC_50_ of 0.03 mm–0.96 mm against *Onchocerca ochengi* microfilariae, *O. ochengi* adults, wild-type *C. elegans* and anthelmintic-resistant strains of *C. elegans, as well as its much higher thresholds revealed by in vivo toxicity data, suggested the potential of ellagic acid as a therapeutic option for nematode infections, including cases of antihelmintic resistance* (Ndjonka et al., 2014)

Artemisinins are among the most potent antimalarials with excellent safety and tolerability profile but unfortunately, artemisinin resistance is common in Southeast Asia (Rosenthal, 2018). Treatment with the whole plant of *Artemisia annua* L. [Asteraceae] (where artemisinin can be derived) was found to be more effective and more resilient than the pure artemisinin to the evolution of parasite resistance. This may be due to the enhanced bioavailability of artemisinin, synergism among the constituents including artemisinin and the presence of other phytochemicals with antimalarial activity independently of artemisinin (Elfawal et al., 2015).

## Patents for available plant products in tackling AMR

Several compounds isolated from plants have been patented for their extensive antimicrobial activities in combating drug-resistant microorganisms. Artonin I, a flavonoid isolated from a tropical plant *Morus mesozygia* Stapf [Moraceae], was patented as a potent inhibitor of all tested strains of MDR *S. aureus* (EMRSA-17, EMRSA-16, MRSA-252 and clinical isolates) through high throughput screening method (Microplate Alamar Blue Assay (MABA)) employed (Rahman, 2014). Its antimicrobial effect was found to be executed by increasing the sensitivity of MDR bacteria to drugs through the MDR efflux pump inhibition and also causing damage in the MDR *S. aureus* cells through the generation of reactive oxygen species (O_2_^−^). The combination of Artonin I with several conventional antibiotics tested (e.g. ciprofloxacin, clindamycin, chloramphenicol) showed substantial synergistic action with >1000 fold reduction in the MIC value as compared to single antibiotics used, indicating a great potential in overcoming the rising incidence of MDR *Staphylococcus* infections (Rahman, 2014). Besides, the bioactive compound isolated from the dry root of *Scutellaria baicalensis* Georgi [Lamiaceae], namely baicalin, was patented for its inhibitory effect on *A. baumannii*, *Stenotrophomonas maltophilia* and *E. coli* containing NDM-1 drug resistance gene, leading to a discovery that has potential to contribute to the treatment and/or prevention of infections caused by pathogens with antimicrobial resistance. As for fungal and parasitic infections, biologically active extracts from *Aframomum aulacocarpos* Pellegr. ex Koechlin [Zingiberaceae], *Aframomum daniellii* (Hook.f.) K.Schum. [Zingiberaceae], *Dracaena arborea* (Willd.) Link [Asparagaceae], *Eupatorium odoratum* L. [Asteraceae], *Glossocalyx brevipes* Benth. [Monimiaceae] and *Napoleonaea imperialis* P. Beauv. [Lecythidaceae] were patented as a treatment choice for drug-resistant fungal and protozoa-related diseases (Okunji et al., 2006).

## Limitations

The review provided a general perspective on the drug resistance of all the microbes and the search strategy was limited to open-access journals and government databases. However, all the recent articles related to the topic have been included in this review. In addition, despite the fact that microbes are less likely to develop resistance towards natural products as compared to conventional antimicrobial agents, recent studies have identified insusceptibility or resistance of microbes towards natural extracts and compounds. For instance, Fagbemi *et al.* (Ferdinand et al., 2009) discovered that *E. coli* ATCC 25922, *E. coli, P. aeruginosa, Salmonella paratyphi, Shigella fleneri* were resistant to the aqueous extract of *Musa sapientum* L. [Musaceae], *Cymbopogon citratus* (DC.) Stapf [Poaceae] and *Curcuma longa* L. [Zingiberaceae]. Consistently, *P. aeruginosa* was also found to be highly resistant to *C. citratus* oil in another independent study (Naik et al., 2010). Furthermore, drug-resistant strains have been isolated from plant products which could be introduced to the patients who consumed these plant products. Ceftriaxone- and tetracycline-resistant bacteria were isolated from organic garlic, traditional onion, organic onion powder and turmeric samples (Brown and Jiang, 2008). Nevertheless, it was revealed that AMR could have been developed as a consequence of adaptation to environmental stresses such as nutrient limitation, oxidative stress and the exposure of the microbes to certain temperature, pH and osmotic pressure (Huang et al., 2021). Hence, it is important to have a thorough understanding of the factors contributing to AMR before introducing these plant products as an alternative for the management of AMR.

## Conclusion

Antimicrobial resistance (AMR) requires serious attention due to the increasing origin of various infections caused by bacteria, viruses, fungi and parasites. Most of the currently available antimicrobials, including antibiotics, antivirals, antifungals and antiparasitics, were unable to effectively treat infections caused by pathogens with AMR. Thus, it is crucial to identify and search for more potential treatments from plant secondary metabolites. In conclusion, we expect several combinations of secondary metabolites may effectively treat AMR-related infections.
