# Supplementary material for: Review on Plant-Based Management in Combating Antimicrobial Resistance - Mechanistic Perspective
Source: Front Pharmacol. 2022 Sep 29;13:879495. doi: 10.3389/fphar.2022.879495 (PMC9557208; doi:10.3389/fphar.2022.879495)
Supplement: Supplementary file 1 [file Table1.docx]

**Supplementary Table 1: Plants and their secondary metabolites with reported antibacterial activity against various drug-resistant bacteria.**

| Plants | Reported secondary metabolites | Mechanism of action | Bacteria | Assay method | Reference |
| --- | --- | --- | --- | --- | --- |
| - | Caffeine **(I)** | QS inhibition | *P. aeruginosa* | Chromobacterium violaceum CV026 biosensor (agar diffusion) | (Norizan et al., 2013) |
| - | Eugenol **(II)** | Reduce expression of major exotoxin  Anti-biofilm activity | MRSA | TNF release assay, hemolysin assay, RT-PCR analysis | (Yadav et al., 2015) |
| *Adansonia digitata* L. [Malvaceae] | Phenols  Polyphenols  Saponins  Tannins  Sterols  Triterpenes | - | MDR *E. coli*  MDR *E. aerogenes*  MDR *E. cloacae*  MDR *K. pneumoniae*  MDR *P. stuartii* | MIC & MBC model | (Djeussi et al., 2013) |
| *Adiantum capillus-veneris* L. [Pteridaceae] | - | Quorum quenching against *S. aureus* | MDR *E. faecium*  MDR *S. aureus* | Broth microdilution testing, biofilm inhibition assay | (Khan et al., 2018) |
| *Aframomum alboviolaceum* (Ridl.) K.Schum. [Zingiberaceae] | Alkaloids  Anthocyanins  Flavonoids  Phenols  Polyphenols  Triterpenes | - | MDR *E. coli*  MDR *E. aerogenes*  MDR *E. cloacae*  MDR *K. pneumoniae*  MDR *P. stuartii* | MIC & MBC model | (Djeussi et al., 2013) |
| *Aframomum polyanthum* (K.Schum.) K.Schum. [Zingiberaceae] | Anthocyanins  Phenols  Polyphenols  Saponins  Triterpenes | - | MDR *E. coli*  MDR *E. aerogenes*  MDR *E. cloacae*  MDR *K. pneumonia*  MDR *P. stuartii* | MIC & MBC model | (Djeussi et al., 2013) |
| *Alkanna tinctoria* (L.) Tausch [Boraginaceae] | Alkaloids  Flavonoids  Carbohydrates | Efflux pump inhibition | *E. coli*  *A. baumannii*  *P. aeruginosa* | MIC & MBC model | (Khan et al., 2015) |
| *Anogeissus acuminata* DC. [Combretaceae] | Anolignan B  Conocarpan  Dihydrodehydrodiconiferylalcohol*  Alkaloids  Glycosides  Terpenoids  Saponins  Tannins  Flavonoids  Steroids  Anthraquinones | Anti-biofilm activity | *E. faecalis*  *S. aureus*  *A. baumannii*  *C. freundii*  *E. aerogenes*  *E. coli*  *K. oxytoca*  *K. pneumoniae*  *P. mirabilis*  *P. vulgaris*  *P. aeruginosa* | Agar well diffusion method | (Mishra et al., 2017) |
| *Anonidium mannii* (Oliv.) Engl. & Diels [Annonaceae] | Alkaloids  Phenols  Polyphenols  Saponins  Tannins  Sterols  Triterpenes | - | MDR *E. coli*  MDR *E. aerogenes*  MDR *E. cloacae*  MDR *K. pneumoniae* | MIC & MBC model | (Djeussi et al., 2013) |
| *Artemisia absinthium* L. [Asteraceae] | - | Quorum quenching against *S. aureus* | MDR *S. aureus* | Broth microdilution testing, biofilm inhibition assay | (Khan et al., 2018) |
| *Azadirachta indica* A.Juss. [Meliaceae] | Mahmoodin*  Resins  Glycosides  Terpenoids  Saponins  Steroids | QS inhibition  Anti-biofilm activity | *E. faecalis*  *S. aureus*  *A. baumannii*  *C. freundii*  *E. aerogenes*  *E. coli*  *K. oxytoca*  *K. pneumoniae*  *P. mirabilis*  *P. vulgaris*  *P. aeruginosa* | Agar well diffusion method | (Vikram et al., 2010; Mishra et al., 2017) |
| *Bauhinia variegata* L. [Fabaceae] | Kaempferol*  Alkaloids  Glycosides  Saponins  Tannins  Steroids  Anthraquinones | Cell membrane disruption | *E. faecalis*  *S. aureus*  *A. baumannii*  *C. freundii*  *E. aerogenes*  *E. coli*  *K. oxytoca*  *K. pneumoniae*  *P. mirabilis*  *P. vulgaris*  *P. aeruginosa* | Agar well diffusion method | (Mishra et al., 2013, 2017) |
| *Boerhavia diffusa* L. [Nyctaginaceae] | Ursolic acid*  Alkaloids  Tannins  Flavonoids  Steroids  Anthraquinones | Disruption of cell wall permeability | *E. faecalis*  *S. aureus*  *A. baumannii*  *C. freundii*  *E. aerogenes*  *E. coli*  *K. oxytoca*  *K. pneumoniae*  *P. aeruginosa* | Agar well diffusion method | (Mishra et al., 2017) |
| *Persea lingue* (Ruiz & Pav.) Nees [Lauraceae] | Kaempferol **(III)** | NorA efflux pump inhibition | MRSA  *P. aeruginosa* | MIC model | (Holler et al., 2012) |
| *Callistemon citrinus* L. [Myrtaceae] | Alkaloids | Efflux pump inhibition | MRSA  *P. aeruginosa* | MIC model | (Mabhiza et al., 2016) |
| *Chamaemelum nobile* (L.) All. [Asteraceae] | - | QS inhibition, anti-biofilm activity | *P. aeruginosa* | MIC model, biofilm inhibition concentration (6.25–25 mg/mL) | (Kazemian et al., 2015) |
| *Chelidonium majus* L. [Papaveraceae] | Sanguinarine **(IV)** | Prevent bacterial cell wall division | MRSA | MIC model (1.9 mg/mL) | (Zielinska et al., 2019) |
| *Cinnamomum tamala* (Buch.-Ham.) T.Nees & C.H.Eberm. [Lauraceae] | Cinnamaldehyde **(V)** | Disruption of membrane integrity | MDR *H. pylori* | *In-vitro* model | (Thapa et al., 2018) |
| *Croton macrostachyus* Hochst. ex Delile [Euphorbiaceae] | Triterpenes  Sterols, polyphenols  Saponins | Efflux pump inhibition | MRSA | MIC & MBC by broth microdilution method | (Voukeng et al., 2016) |
| *Erythrina sigmoidea* Hua [Fabaceae] | Neobavaisoflavone*  Atalantoflavone  Bidwillon A  Neocyclomorucin  6α-hydroxyphaseollidin  Sigmoidin I | ­- | MDR *E. coli*  MDR *E. aerogenes*  MDR *E. cloacae*  MDR *K. pneumoniae*  MDR *P. stuartii* |  | (Djeussi et al., 2015) |
| *Hibiscus sabdariffa* L. [Malvaceae] | Alkaloids  Flavonoids  Phenols  Polyphenols  Saponins  Sterols  Triterpenes | - | MDR *E. coli*  MDR *E. aerogenes*  MDR *E. cloacae*  MDR *K. pneumoniae*  MDR *P. stuartii* | MIC & MBC model | (Djeussi et al., 2013) |
| *Kalanchoe blossfeldiana* Poelln. [Crassulaceae] | Methanolic extract | QS-mediated biofilm formation | *P. aeruginosa* | MIC model, laser scanning microscopy | (Sarkar et al., 2015) |
| *Martynia annua* L. [Martyniaceae] | - | - | MDR *A. baumannii*  MDR *E. faecium*  MDR *K. pneumoniae*  MDR *S. aureus* | Broth microdilution testing, biofilm inhibition assay | (Khan et al., 2018) |
| *Matricaria chamomilla* L. [Asteraceae] | - | - | MDR *E. coli*  MDR *Klebsiella* spp.  MDR *P. aeruginosa*  MDR *P. mirabilis*  MDR *S. aureus* | Agar well diffusion method, MIC & MBC model | (Atef et al., 2019) |
| *Moringa oleifera* Lam. [Moringaceae] | Alkaloids  Polyphenols  Flaconoids  Anthraquinones  Coumarins  Tannins  Triterpenes  Sterols  Saponins | - | MDR *E. coli*  MDR *Klebsiella* spp.  MDR *P. aeruginosa*  MDR *P. mirabilis*  MDR *S. aureus* | Agar well diffusion method, MIC &MBC model | (Atef et al., 2019) |
| *Ocimum basilicum* L. [Lamiaceae] | Phytol **(VI)**  Cadinene **(VII)** | Induction of oxidative stress | *A. baumannii*  *E. coli* | MIC by broth microdilution technique | (De Assis et al., 2018) |
| *Ocimum gratissimum* L. [Lamiaceae] | Alkaloids  Phenols  Polyphenols  Tannins  Sterols  Triterpenes | - | MDR *E. coli*  MDR *E. aerogenes*  MDR *E. cloacae*  MDR *K. pneumoniae*  MDR *P. stuartii* | MIC & MBC model | (Djeussi et al., 2013) |
| *Olea europaea* L. [Oleaceae] | Oleanolic acid **(VIII)** | Inhibition of efflux pump | *P. aeruginosa*  *S. pneumoniae*  *M. tuberculosis*  *E. coli* | MIC model | (Jesus et al., 2015) |
| *Piper regnellii* (Miq.) C.DC. [Piperaceae] | Conocarpan  Eupomathenoid-5 | Anti-biofilm activity | MRSA  MSSA | MIC model, biofilm assay | (Brambilla et al., 2017) |
| *Punica granatum* L. [Lythraceae] | Stigmasterol*  Alkaloids  Glycosides  Terpenoids  Saponins  Tannins  Flavonoids  Steroids  Anthraquinones | Inhibition of bacterial enzyme | *E. faecalis*  *S. aureus*  *A. baumannii*  *C. freundii*  *E. aerogenes*  *E. coli*  *K. oxytoca*  *K. pneumoniae*  *P. mirabilis*  *P. vulgaris*  *P. aeruginosa* | Agar well diffusion method | (Mishra et al., 2017; Alawode et al., 2021) |
| *Scutellaria baicalensis* Georgi [Lamiaceae] | Baicalein (IX) | Efflux pump inhibition  Inhibition of bacterial cell wall synthesis | MRSA  *E. coli*  VRE | MIC model, time-kill study | (Fujita et al., 2005) |
| *Soymida febrifuga* (Roxb.) A.Juss. [Meliaceae] | Luteolin-7-O-glucoside*  Methyl angolensate  Alkaloids  Resins  Glycosides  Terpenoids  Tannins  Flavonoids  Steroids,  Anthraquinones | Cell membrane disruption | *E. faecalis*  *S. aureus*  *A. baumannii*  *C. freundii*  *E. aerogenes*  *E. coli*  *K. oxytoca*  *K. pneumoniae*  *P. mirabilis*  *P. vulgaris*  *P. aeruginosa* | Agar well diffusion method, disc diffusion method | (Mishra et al., 2017) (Chiruvella et al., 2007) |
| *Swertia chirata* Buch.-Ham. ex C.B. Clarke, [Gentianaceae] | - | Quorum quenching against *S. aureus* | MDR *S. aureus* | Broth microdilution testing, biofilm inhibition assay | (Khan et al., 2018) |
| *Tamarindus indica* L. [Fabaceae] | Alkaloids  Flavonoids  Phenols  Polyphenols  Saponins  Sterols  Triterpenes | - | MDR *E. coli*  MDR *E. aerogenes*  MDR *E. cloacae*  MDR *K. pneumoniae* | MIC & MBC model | (Djeussi et al., 2013) |
| *Terminalia chebula* Retz. [Combretaceae] | Arjungenin*  Alkaloids  Resins  Glycosides  Terpenoids  Tannins  Flavonoids  Anthraquinones | Anti-biofilm activity | *E. faecalis*  *S. aureus*  *A. baumannii*  *E. coli*  *K. oxytoca*  *K. pneumoniae*  *P. mirabilis*  *P. aeruginosa* | Agar well diffusion method | (Mishra et al., 2017) |
| *Thymbra spicata* L. [Lamiaceae] | Carvacrol (X)  Thymol (XI)  Camphor | Efflux pump inhibition | *E. coli* | MIC & MBC microdilution method | (Abdallah and Omar, 2019) |
| *Tinospora cordifolia* (Willd.) Hook.f. & Thomson [Menispermaceae] | Berberine*  Quinones  Polyphenols  Alkaloids  Flavonoids  Tannins  Coumarins  Terpenoids  Lectins  Glycosides  Saponins  Steroids | Inhibition of bacterial enzyme  Cell membrane disruption | *E. faecalis*  *S. aureus*  *C. freundii*  *E. aerogenes*  *E. coli*  *K. oxytoca*  *K. pneumoniae*  *P. mirabilis*  *P. aeruginosa* | Agar well diffusion method | (Mishra et al., 2017; Agarwal et al., 2019) |
| *Tribulus terrestris* L. [Zygophyllaceae] | Quercetin  Terpenoids  Steroids | Cell membrane disruption | *E. faecalis*  *S. aureus*  *A. baumannii*  *C. freundii*  *E. coli*  *K. oxytoca*  *K. pneumoniae*  *P. mirabilis*  *P. vulgaris*  *P. aeruginosa* | Agar well diffusion method | (Mishra et al., 2017) |
| *Zanthoxylum armatum* DC. [Rutaceae] | threo-3-methoxy-5-hydroxy-phenylpropanetriol-8-O-β-D-glucopyranoside  3-[[6-O-(6-deoxy-α-L-mannopyranosyl)-β-D-glucopyranosyl]oxy]-2-(3,4-dihydroxyphenyl)-5,7-dihydroxy-4H-1-benzopyran-4-one  6′-methoxy-(8α,9R)-cinchonan-9-ol  N-(2,3-dihydroxy-2-methylpropyl)-2,6,8,10-dodecatetraenamide  3,5,7-trihydroxy-8-methoxy-2-(4-methoxyphenyl)-4H-1-benzopyran-4-one  N-(2-methylpropyl)-2,6,8,10-dodecatetraenamide  N-(2-methylpropyl)-2,4,8,10,12-tetradecapentaenamide  9,12,15-octadecatrienoic acid | Quorum quenching against *S. aureus* | MDR *E. faecium*  MDR *S. aureus* | Broth microdilution testing, biofilm inhibition assay | (Khan et al., 2018) |
| *Zingiber officinale* Roscoe [Zingiberaceae] | Lariciresinol | Efflux pump inhibition | *S. typhimurium* | Agar well diffusion method, MIC microdilution method | (Mehta et al., 2022) |

**References**

Abdallah, L., and Omar, G. (2019). Antibacterial effect of some wild medicinal plants in Palestine against multidrug resistant Escherichia coli clinical isolate. *Brazilian J. Biol. Sci.* 6, 103–113. doi:10.21472/bjbs.061209.

Agarwal, S., Ramamurthy, P., Fernandes, B., Rath, A., and Sidhu, P. (2019). Assessment of antimicrobial activity of different concentrations of Tinospora cordifolia against Streptococcus mutans: An in vitro study. *Dent. Res. J. (Isfahan).* 16, 24–28. doi:10.4103/1735-3327.249556.

Alawode, T. T., Lajide, L., Olaleye, M., and Owolabi, B. (2021). Stigmasterol and β-Sitosterol: Antimicrobial Compounds in the Leaves of Icacina trichantha identified by GC–MS. *Beni-Suef Univ. J. Basic Appl. Sci.* 10, 1–8. doi:10.1186/s43088-021-00170-3.

Atef, N. M., Shanab, S. M., Negm, S. I., and Abbas, Y. A. (2019). Evaluation of antimicrobial activity of some plant extracts against antibiotic susceptible and resistant bacterial strains causing wound infection. *Bull. Natl. Res. Cent.* 43, 1–11. doi:10.1186/s42269-019-0184-9.

Brambilla, L. Z. S., Endo, E. H., Cortez, D. A. G., and Filho, B. P. D. (2017). Anti-biofilm activity against staphylococcus aureus mrsa and mssa of neolignans and extract of piper regnellii. *Rev. Bras. Farmacogn.* 27, 112–117. doi:10.1016/j.bjp.2016.08.008.

Chiruvella, K. K., Mohammed, A., Dampuri, G., Ghanta, R. G., and Raghavan, S. C. (2007). Phytochemical and Antimicrobial Studies of Methyl Angolensate and Luteolin-7-O-glucoside Isolated from Callus Cultures of Soymida febrifuga. *Int. J. Biomed. Sci.* 3, 269–78. Available at: /pmc/articles/PMC3614660/ [Accessed February 13, 2022].

De Assis, F. V., Siqueira, F. L., Gonçalves, I. E., Lacerda, R. P., Nascimento, R. A., Araújo, S. G., et al. (2018). Antibacterial activity of Lamiaceae plant extracts in clinical isolates of multidrug-resistant bacteria. *An. Acad. Bras. Cienc.* 90, 1665–1670. doi:10.1590/0001-3765201820160870.

Djeussi, D. E., Noumedem, J. A. K., Seukep, J. A., Fankam, A. G., Voukeng, I. K., Tankeo, S. B., et al. (2013). Antibacterial activities of selected edible plants extracts against multidrug-resistant Gram-negative bacteria. *BMC Complement. Altern. Med.* 13, 1–8. doi:10.1186/1472-6882-13-164/TABLES/3.

Djeussi, D. E., Sandjo, L. P., Noumedem, J. A. K., Omosa, L. K., Ngadjui, B. T., and Kuete, V. (2015). Antibacterial activities of the methanol extracts and compounds from Erythrina sigmoidea against Gram-negative multi-drug resistant phenotypes. *BMC Complement. Altern. Med.* 15. doi:10.1186/s12906-015-0978-8.

Fujita, M., Shiota, S., Kuroda, T., Hatano, T., Yoshida, T., Mizushima, T., et al. (2005). Remarkable Synergies between Baicalein and Tetracycline, and Baicalein and β-Lactams against Methicillin-Resistant Staphylococcus aureus. *Microbiol. Immunol.* 49, 391–396. doi:10.1111/j.1348-0421.2005.tb03732.x.

Holler, J. G., Christensen, S. B., Slotved, H.-C., Rasmussen, H. B., Gúzman, A., Olsen, C.-E., et al. (2012). Novel inhibitory activity of the Staphylococcus aureus NorA efflux pump by a kaempferol rhamnoside isolated from Persea lingue Nees. *J. Antimicrob. Chemother.* 67, 1138–1144. doi:10.1093/jac/dks005.

Jesus, J. A., Lago, J. H. G., Laurenti, M. D., Yamamoto, E. S., and Passero, L. F. D. (2015). Antimicrobial activity of oleanolic and ursolic acids: an update. *Evid. Based. Complement. Alternat. Med.* 2015, 620472. doi:10.1155/2015/620472.

Kazemian, H., Ghafourian, S., Heidari, H., Amiri, P., Yamchi, J. K., Shavalipour, A., et al. (2015). Antibacterial, anti-swarming and anti-biofilm formation activities of Chamaemelum nobile against Pseudomonas aeruginosa. *Rev. Soc. Bras. Med. Trop.* 48, 432–436. doi:10.1590/0037-8682-0065-2015.

Khan, M. F., Tang, H., Lyles, J. T., Pineau, R., Mashwani, Z. ur R., and Quave, C. L. (2018). Antibacterial properties of medicinal plants from Pakistan against multidrug-resistant ESKAPE pathogens. *Front. Pharmacol.* 9. doi:10.3389/fphar.2018.00815.

Khan, U. A., Rahman, H., Qasim, M., Hussain, A., Azizllah, A., Murad, W., et al. (2015). Alkanna tinctoria leaves extracts: A prospective remedy against multidrug resistant human pathogenic bacteria. *BMC Complement. Altern. Med.* 15, 1–6. doi:10.1186/s12906-015-0646-z.

Mabhiza, D., Chitemerere, T., and Mukanganyama, S. (2016). Antibacterial Properties of Alkaloid Extracts from *Callistemon citrinus* and *Vernonia adoensis* against *Staphylococcus aureus* and *Pseudomonas aeruginosa*. *Int. J. Med. Chem.* 2016, 6304163. doi:10.1155/2016/6304163.

Mehta, J., Rolta, R., and Dev, K. (2022). Role of medicinal plants from North Western Himalayas as an efflux pump inhibitor against MDR AcrAB-TolC Salmonella enterica serovar typhimurium: In vitro and In silico studies. *J. Ethnopharmacol.* 282, 114589. doi:10.1016/j.jep.2021.114589.

Mishra, A., Sharma, A. K., Kumar, S., Saxena, A. K., and Pandey, A. K. (2013). Bauhinia variegata leaf extracts exhibit considerable antibacterial, antioxidant, and anticancer activities. *Biomed Res. Int.* 2013. doi:10.1155/2013/915436.

Mishra, M. P., Rath, S., Swain, S. S., Ghosh, G., Das, D., and Padhy, R. N. (2017). In vitro antibacterial activity of crude extracts of 9 selected medicinal plants against UTI causing MDR bacteria. *J. King Saud Univ. - Sci.* 29, 84–95. doi:10.1016/j.jksus.2015.05.007.

Norizan, S. N., Yin, W.-F., and Chan, K.-G. (2013). Caffeine as a Potential Quorum Sensing Inhibitor. *Sensors*  13. doi:10.3390/s130405117.

Sarkar, R., Mondal, C., Bera, R., Chakraborty, S., Barik, R., Roy, P., et al. (2015). Antimicrobial properties of Kalanchoe blossfeldiana: a focus on drug resistance with particular reference to quorum sensing-mediated bacterial biofilm formation. *J. Pharm. Pharmacol.* 67, 951–962. doi:10.1111/jphp.12397.

Thapa, B., Singh, A., and Tuladhar, R. (2018). In vitro Antibacterial Effect of Medicinal Plants Against Multidrug Resistant Gram Negative Bacteria. *Tribhuvan Univ. J. Microbiol.* 5, 25–31. doi:10.3126/tujm.v5i0.22298.

Vikram, A., Jesudhasan, P. R., Jayaprakasha, G. K., Pillai, B. S., and Patil, B. S. (2010). Grapefruit bioactive limonoids modulate E. coli O157:H7 TTSS and biofilm. *Int. J. Food Microbiol.* 140, 109–116. doi:10.1016/j.ijfoodmicro.2010.04.012.

Voukeng, I. K., Beng, V. P., and Kuete, V. (2016). Antibacterial activity of six medicinal Cameroonian plants against Gram-positive and Gram-negative multidrug resistant phenotypes. *BMC Complement. Altern. Med.* 16. doi:10.1186/s12906-016-1371-y.

Yadav, M. K., Chae, S.-W., Im, G. J., Chung, J.-W., and Song, J.-J. (2015). Eugenol: a phyto-compound effective against methicillin-resistant and methicillin-sensitive Staphylococcus aureus clinical strain biofilms. *PLoS One* 10, e0119564–e0119564. doi:10.1371/journal.pone.0119564.

Zielinska, S., Wójciak-Kosior, M., Dziagwa-Becker, M., Glensk, M., Sowa, I., Fijalkowski, K., et al. (2019). The activity of isoquinoline alkaloids and extracts from chelidonium majus against pathogenic bacteria and Candida sp. *Toxins (Basel).* 11, 1–13. doi:10.3390/toxins11070406.
